# The Ty1 retrotransposon harbors a DNA region that performs dual functions as both a gene silencing and chromatin insulator

**DOI:** 10.1038/s41598-024-67242-z

**Published:** 2024-07-18

**Authors:** Hiroshi Masumoto, Hideki Muto, Koichi Yano, Yohei Kurosaki, Hironori Niki

**Affiliations:** 1 Biomedical Research Support Center (BRSC), Nagasaki University School of Medicine, 1-12-4 Sakamoto, Nagasaki, Nagasaki 852-8523 Japan; 2 Microbial Physiology Laboratory, Department of Gene Function and Phenomics, National Institute of Genetics, 1,111 Yata, Mishima, Shizuoka 411-8540 Japan; 3 National Research Center for the Control and Prevention of Infectious Diseases, Nagasaki University, 1-12-4 Sakamoto, Nagasaki, Nagasaki 852-8523 Japan; 4 Present Address: Department of Life Science, College of Science, Rikkyo University, Tokyo, 171-8501 Japan

**Keywords:** Chromatin structure, Gene silencing

## Abstract

In various eukaryotic kingdoms, long terminal repeat (LTR) retrotransposons repress transcription by infiltrating heterochromatin generated within their elements. In contrast, the budding yeast LTR retrotransposon Ty1 does not itself undergo transcriptional repression, although it is capable of repressing the transcription of the inserted genes within it. In this study, we identified a DNA region within Ty1 that exerts its silencing effect via sequence orientation. We identified a DNA region within the Ty1 group-specific antigen (GAG) gene that causes gene silencing, termed GAG silencing (GAGsi), in which the silent chromatin in the GAGsi region is created by euchromatin-specific histone modifications. A characteristic inverted repeat (IR) sequence is present at the 5' end of this region, forming a chromatin boundary between promoter-specific chromatin upstream of the IR sequence and silent chromatin downstream of the IR sequence. In addition, Esc2 and Rad57, which are involved in DNA repair, were required for GAGsi silencing. Finally, the chromatin boundary was required for the transcription of Ty1 itself. Thus, the GAGsi sequence contributes to the creation of a chromatin environment that promotes Ty1 transcription.

## Introduction

**Figure 1:**
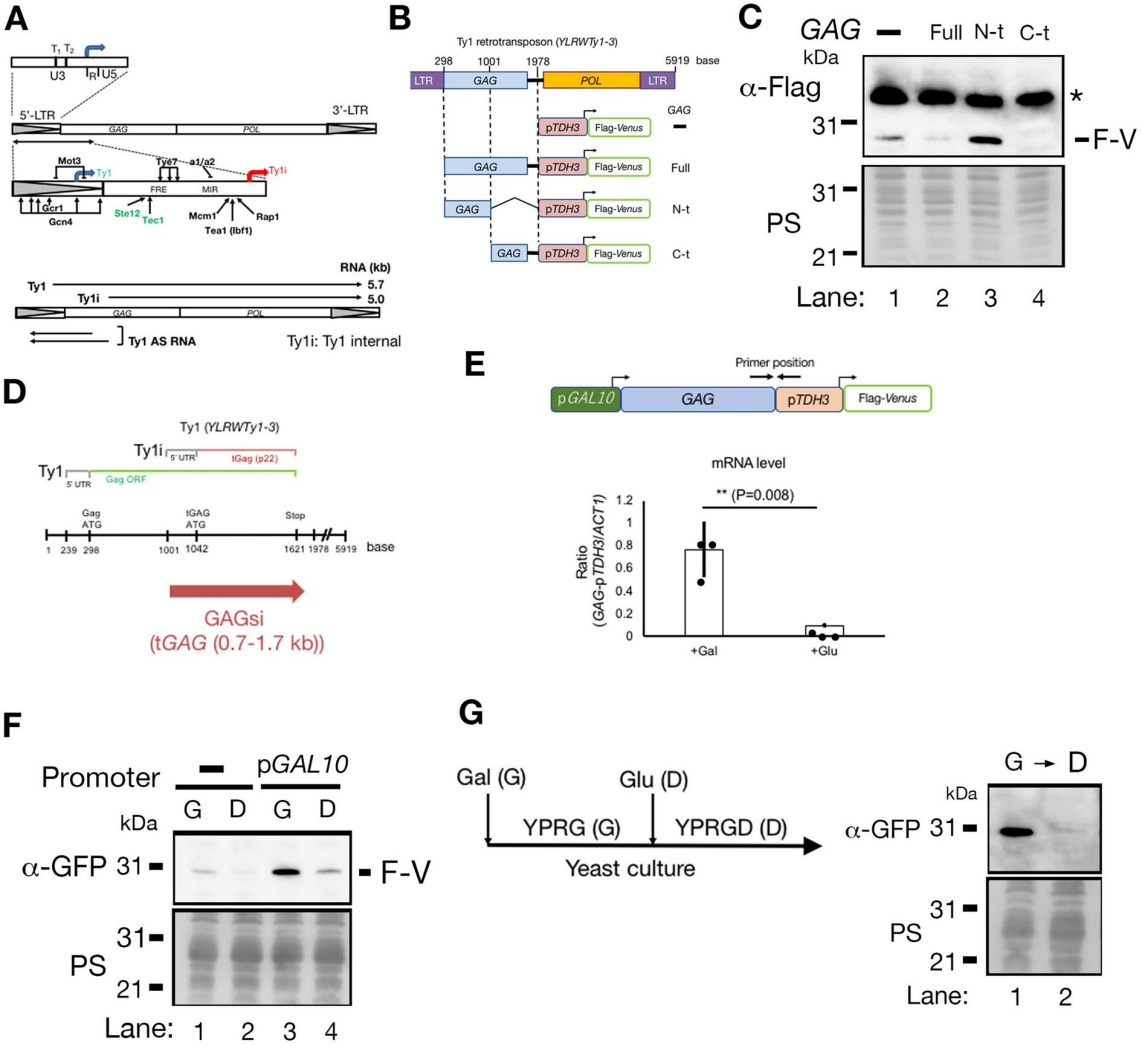
The GAGsi region creates gene silencing. (**A**) Scheme of the transcriptional regulation sites of Ty1 and two types of Ty1 transcripts. The data were adapted from Curcio et al.^3^. (**B**) Identification of the sequence responsible for silencing inside the *GAG* gene. Illustration of each size truncated *GAG* gene fragment connected to the *TDH3* promoter-Flag-Venus. (**C**) Immunoblot comparing the expression levels of Flag-epitope tagging Venus (F-V), a variant of yellow fluorescent protein (YFP), in each construct illustrated in (**B**). Anti-Flag epitope antibody was used in the immunoblot. PS: Ponceau S staining to detect the proteins in each lane as loading controls. *: non-specific band. (**D**) The position of the GAGsi region in the Ty1 element. *YLRWTy1-3* sequence is a representative Ty1 element used in this study. (**E**) Comparison of mRNA transcription levels. The *GAL1* promoter (p*GAL1*) connected to the GAGsi-*TDH3* promoter (p*TDH3*)-Flag-*Venus* (top); GAG-p*TDH3*: primer positions used in RT-PCR. mRNA transcription level comparison (bottom). + Gal: galactose used in the culture medium. + Glu: glucose used in the culture medium. The results are the average ± s. d. (n = 3, independent experiments) and analyzed by unpaired *t*-test (two-tailed). (**F**) Immunoblot comparing the expression levels of Flag tagging Venus (F-V) either in the presence of galactose (G) or glucose (D) used in (**E**). Anti-GFP antibody was used in the immunoblot. (**G**) Time course of yeast culture exchange from YPRG medium to YPRGD medium (left scheme). YPRG: yeast extract (Y)-polypeptone (P)-raffinose (R)-galactose (G). YPRGD: YPRG with glucose (**D**) Immunoblot of Flag tagging Venus (right). G → D: After culturing in YPRG, glucose was added to the culture medium.

The LTR-retrotransposon moves by the so-called “copy and paste” mechanism, which involves reverse transcription of an RNA intermediate and subsequent integration as an additional copy within the host genome (reviewed in^1,2^). LTR retrotransposons are widespread in the eukaryotic kingdom and share common ancestors with retroviruses. Many LTR retrotransposons contain GAG (group-specific antigen), pro (protease), and pol (polymerase) genes that are derived from exogenous retroviruses. Five families of LTR retrotransposons, Ty1 to Ty5, are the transposable elements in *S. cerevisiae* genomes (review in^3^). Many types of retrotransposons that exist in various eukaryotes are transcriptionally silenced by heterochromatin formation^1,4^. However, budding yeast retrotransposons are actively transcribed, accounting for 0.1% to 0.8% of total cellular RNA and 10% of total polyadenylated mRNA^5,6^. Similar to retroviruses, several cellular stresses, such as adenine depletion or DNA damage agents, induce Ty1 expression^7–9^. Ty1 transcription initiates at the 5’-LTR promoter (Fig. 1A). The U3 region of the 5’-LTR contains promoter sequences recognized by DNA-binding transcriptional activators, as well as TATA box sequences (T_1_ and T_2_) (Fig. 1A)^3,10^. The full length of Ty1 transcript (5.7 kb) contains two partially overlapping open reading frames, *GAG* and *POL* (Fig. 1A)^11^. Ty1 elements also produce a second transcript that initiates at + 1000 bp in the Ty1 element 762 bp downstream of the Ty1 transcription start site (TSS) (Fig. 1A)^10^. This short transcript, termed Ty1 internal (Ty1i), can be observed in *spt3* and *snf5* mutants, in which transcript levels of full-length Ty1 are severely reduced, but are difficult to detect in wild-type cells^12–14^.

Our previous study showed that a gene that was integrated into the position between *GAG* and *POL* genes in the Ty1 element transcriptionally remained repressed^2^. A similar transcriptional silencing mechanism was observed for Ty2. In Ty2-917, a laboratory Ty2 element strain, three regions termed downstream repression sites (DRSs) I, II, and III, which are located in the *GAG* gene, can repress downstream gene transcription downstream^15,16^. Interestingly, the gene silencing observed in Ty1 does not inhibit transcription from the 5’-LTR, limiting the silencing effect unidirectionally. This curious silencing system manifested in Ty1 may be different from retrotransposon silencing by heterochromatin formation in mammals. In this study, we sought to elucidate the gene silencing system within the Ty1 element.

## Results

### The DNA region that controls gene silencing is located within the *GAG* gene in Ty1

A gene that was integrated at the position between the *GAG* gene and the *POL* gene remained transcriptionally silenced^2^. We first split the *GAG* gene sequence into two parts and linked it to the Flag-Venus gene under the *TDH3* promoter (Fig. 1B), and then tested which part was able to repress Flag-Venus expression (Fig. 1C). We used *YLRWTy1-3* sequence as a representative Ty1 element, which is the most expressed Ty1 element^17^. The 3' region (0.7–1.7 kb) of the *GAG* gene (from 1001 to 1976 nt in the Ty1 element) suppressed the expression of the Flag-Venus, whereas the 5' region (0.7 kb) of the *GAG* gene (from 298 to 1000 nt in the Ty1 element) did not (Fig. 1C: lane 2–4). We named the region spanning from the Ty1i TSS to the 3’ end of the *GAG* gene as Ty1 element GAG silencing (GAGsi), covering 1001 to 1976 nt in the Ty1 element (Fig. 1D). To investigate whether the transcriptional fork migration was able to release the gene repression exerted by GAGsi region, we used a yeast strain with the galactose-inducible *GAL10* promoter upstream of the *GAG* gene -*TDH3* promoter-Flag-Venus gene (Fig. 1E: cartoon). Real-time polymerase chain reaction (RT-PCR) confirmed that RNA strands were synthesized at the junction of the *GAG* gene and *TDH3* promoters in the presence of galactose (+ Gal), but not in the presence of glucose (+ Glu) (Fig. 1E: graph). We confirmed that the transcription fork originating from the *GAL10* promoter passed through the GAGsi region and reached the 3' end of Flag-Venus. Using DNA reverse-transcribed from RNA extracted from cells cultured in the presence of galactose, PCR was performed with primers set at the 5' end of GAGsi and the 3' end of Flag-Venus (Fig. S1A: cartoon) and a plasmid containing GAGsi-p*TDH3*-Flag-Venus was used as the PCR control. The region (3.1 kbp) containing GAGsi-p*TDH3*-Flag-Venus could be amplified (Fig. S1B), indicating that the transcription fork starting from the *GAL10* promoter passed through GAGsi and reached the 3' end of Flag-Venus. Although Flag-Venus was repressed in the presence of either galactose or glucose without the *GAL10* promoter (Fig. 1F: lanes 1 and 2), Flag-Venus was expressed in the presence of galactose but not glucose under the *GAL10* promoter (Fig. 1F: lanes 3 and 4). We generated a construct without the *TDH3* promoter domain and tested whether Flag-Venus expression could be induced by activation of the *GAL10* promoter, however, Flag-Venus expression did not occur (Fig. S1C). This indicates that Flag-Venus expression is dependent on the immediately preceding *TDH3* promoter, and that mRNA originating from *the GAL10* promoter was not used to translate Flag-Venus. We further confirmed whether arresting the transcription fork could restore the silencing effect of GAGsi. Yeast cells were cultured in the medium containing galactose, and then cells were released in the presence of glucose (Fig. 1G: cartoon). Although Flag-Venus was detected in galactose, it was not detected after release in glucose (Fig. 1G: lanes 1 and 2). Thus, the silencing effect of GAGsi was reversibly switched by the transcription fork.

### An inverted repeat (IR) sequence is necessary for GAGsi silencing

**Figure 2:**
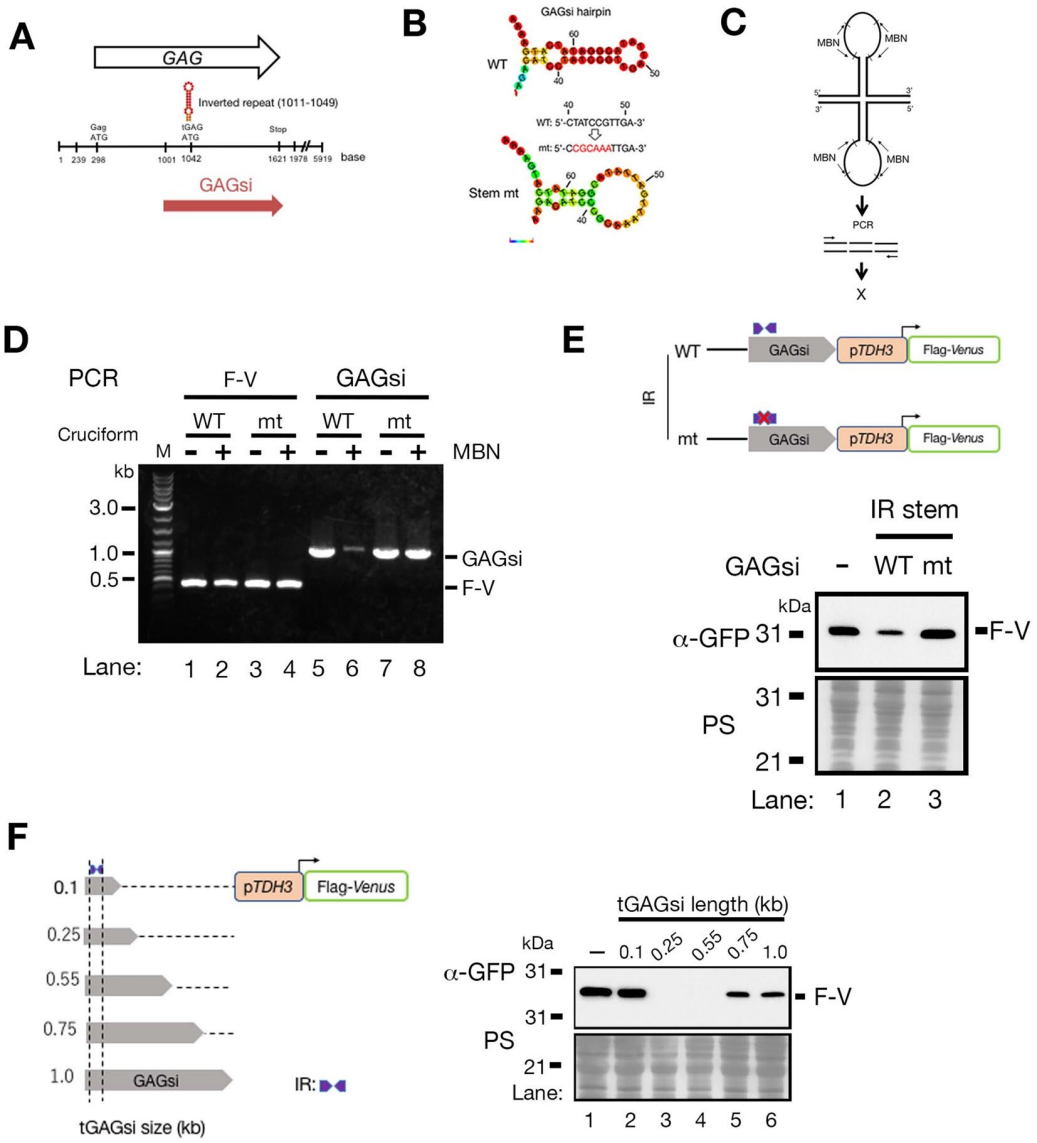
The inverted repeat (IR) sequence in the GAGsi region is necessary for the silencing effect of GAGsi. **(A**) The position of the IR site in GAGsi. (**B**) The probability of IR formation by GAGsi IR wild-type sequence (upper) or the stem sequence mutant (bottom). The base-color gradation from purple to red represents an increase in the probability of IR structure. (**C**) Schematic of Mung-Bean nuclease (MBN)-PCR analysis to detect the IR structure on the plasmid. (**D**) Agarose gel electrophoresis of PCR products with or without post-MBN treatment. F-V: Flag-Venus. (**E**) Immunoblot to compare the expression levels of Flag-Venus between the wild-type and IR-stem mutant. (**F**) GAGsi sequences of various sizes centered at IR sites were connected to p*TDH3*-Flag-Venus (left). Immunoblot comparing the expression levels of FLAG-tagged Venus (F-V) in each construct (right). tGAGsi: truncated GAGsi region. Anti-GFP antibody was used in the immunoblots (**E** and **F**).

The GAGsi region has an IR sequence (1011 to 1049 nt in the Ty1 element) that is perfectly conserved in 28 of total 32 Ty1 elements on the chromosomes registered in the *Saccharomyces* genome database (SGD) (https://www.yeastgenome.org) (Fig. S2A), and that overlaps the first ATG site of Ty1i (Fig. 2A). This IR sequence can form a hairpin structure in free Ty1 mRNA^18^. We also confirmed that this IR sequence was capable of forming a hairpin structure using the RNAfold server (http://rna.tbi.univie.ac.at/cgi-bin/RNAWebSuite/RNAfold.cgi) (Fig. 2B: WT). To determine whether the GAGsi IR sequence is capable of forming hairpin structures in vitro*,* we treated plasmid DNA containing the GAGsi region with mung bean nuclease (MBN) and performed PCR. If MBN can digest the single-stranded DNA (ssDNA) portion on the DNA hairpin structure within the DNA template, the DNA fragment containing the IR sequence is not amplifiable by PCR (Fig. 2C). We confirmed by the RNAfold website that the IR-stem mutant had thermodynamic difficulties in forming a hairpin structure (Fig. 2B: Stem mt). The Flag-Venus region, which had no obvious IR structure, was amplified by PCR with or without MBN treatment (Fig. 2D: lanes 1–4). The GAGsi region with the wild-type IR sequence was not amplified by PCR after MBN treatment (Fig. 2D: lanes 5 and 6), whereas the GAGsi region with the IR-stem mutant was amplified (Fig. 2D: lanes 7 and 8). Next, we investigated whether the IR sequence is important for the silencing effect of the GAGsi because DNA secondary structures such as hairpin structures play an important role in the silencing core region of Line-1, the non-LTR type retrotransposon^19^. The level of Flag-Venus expression was found to be higher in the IR stem mutant compared to the IR wild-type sequence. Moreover, it was almost identical to the control (-GAGsi) (Fig. 2E: lanes 1 to 3). This indicates that the IR sequence in the GAGsi region played a crucial role in gene silencing. We confirmed whether mutations in the loop sequence in the IR of the GAGsi region affected gene silencing. The expression levels of Flag-Venus in two loop sequence mutants (1C (T1027C) and 3C (G1025C, T1027C, A1029C ) ) in the Ty1 element were the same as in the control (-GAGsi), whereas the expression was reduced in the WT loop sequence (Fig. S2B: lanes 1–4). Thus, the IR sequence plays an important role in GAGsi silencing. We then used GAGsi regions of different sizes downstream of the IR sequence to determine which region had the strongest silencing effect. We found that the two short sizes of the GAGsi region (0.25 and 0.55 kb), including the IR, showed similarly strong silencing effects (Fig. 2F: lanes 3 and 4) and weakened with increasing distance from the IR sequence (Fig. 2F: lanes 5 and 6). Unexpectedly, the short 0.1 kb fragment containing the IR sequence did not have a silencing effect (Fig. 2F: lanes 1 and 2). This finding suggests that the silencing effect of GAGsi requires its combination with the IR sequence and the sequence adjacent to the IR.

### The GAGsi region creates silent chromatin together with the euchromatin-specific histone modifications

**Figure 3:**
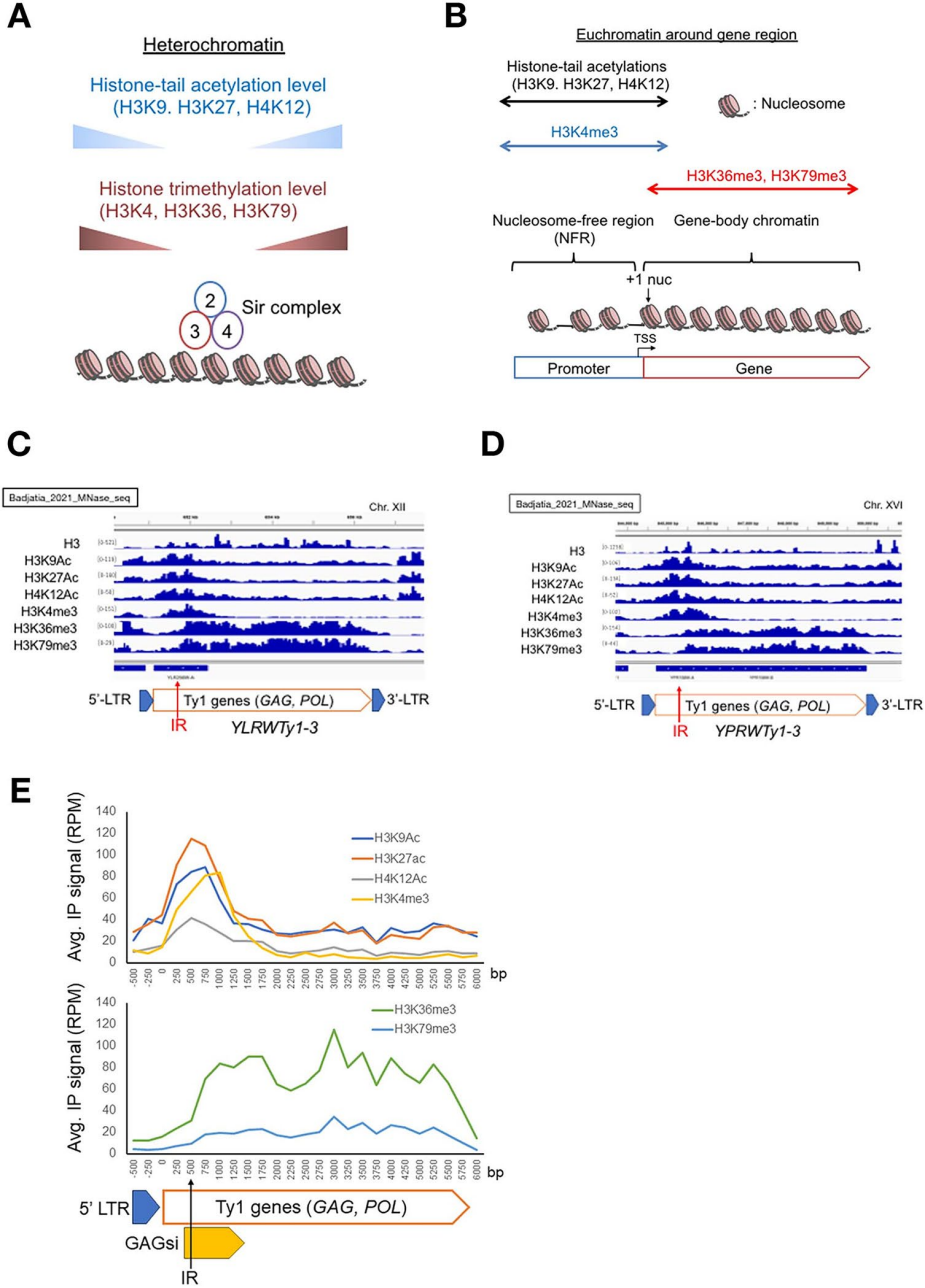
The GAGsi region harbors euchromatin-specific histone modifications and high nucleosome densities. **(A**) Schematic of budding yeast heterochromatin. Sir complex: 2, 3, and 4 represent Sir2, Sir3, and Sir4, respectively. (**B**) Schematic of euchromatin structure around active transcribed gene region. (**C**) and (**D**) MNase-ChIP-seq data monitoring histone H3, histone H3 on lysine 9 (K9) acetylation (H3K9Ac), H3K27Ac, H4K12Ac, H3K4 trimethylation (H3K4me3), H3K36me3 and H3K79me3. The ChIP-seq data adapted from Badjatia et al. (Accession No. GSE151348)^20^ were visualized using Integrative Genomics Viewer (IGV)^71^. Two Ty1 elements, *YLRWTy1-4* and *YGRWTy1-1*, were used (**C** and **D**). (**E**) Occupancy of histone modifications were summed over 31 Ty1 elements by the ChIP-seq data adapted from Badjatia et al. (Accession No. GSE151348)^20^. The *GAG* gene in Ty1 element begins at 0 bp along the X-axis. Black arrowhead: the IR site.

Heterochromatin formation is a major factor in the establishment of gene silencing. To investigate whether the GAGsi region generates the conventional heterochromatin structure within Ty1 elements, we analyzed the histone modification patterns on chromatin by NGS data of the micrococcus nuclease-chromatin immunoprecipitation-sequencing (MNase-ChIP-seq) f already published by Badjatia, N et al. (accession No. GSE151348)^20^. Heterochromatin in *S. cerevisiae* exists in several genomic regions (rDNA, *MAT* loci, *HML*, *HMR* loci, and subtelomere regions) and are composed of low levels of euchromatin-specific histone acetylation (histone H3 on lysine 9 acetylation (H3K9ac), H3K27ac and H4K12ac) and histone trimethylation (H3K4me3, H3K36me3, and H3K79me3) together with heterochromatin-binding proteins such as the Sir2/3/4 complex^21–23^ (Figs. 3A, S3A, and S3B). In contrast, in euchromatin around actively transcribed genes, euchromatin-specific histone acetylation, and H3K4me3 are abundant from the promoter region to the 5' terminus of the gene region (Figs. 3B, S3C, and S3D). H3K36me3 and H3K79me3 are present throughout the gene body region (Figs. 3B, S3C, and S3D). Nucleosome densities decrease at the promoter region, where a nucleosome can slide on the DNA fiber, whereas nucleosome densities increase significantly throughout the gene bodies as gene body chromatin (Figs. 3B, S3C, and S3D). In addition, a well-positioned nucleosome, so-called ' + 1' nucleosome (+ 1 nuc), is located above or just downstream of the TSS (Fig. 3B: arrowhead)^24^.

In thirty-one Ty1 elements other than *YLR035C-A*, in which the *GAG* gene sequence is lost (Fig. S2A), histone acetylation (H3K9ac, H3K27ac and H4K12ac) and H3K4me3 were detected on nucleosomes from 5' regions of Ty1 elements (Fig. 3C–E: Badjatia_2021_Mnase_seq: Accession No.GSE151348). Interestingly, both H3K36me3 and H3K79me3 were detected from regions downstream of IR site in the middle of the *GAG* gene, but not at the TSS position (arrowhead) of the Ty1 element (Fig. 3C-E: Badjatia_2021_Mnase_seq). Considering that the IR site overlaps with the 1st ATG of Ty1i (Fig. 2A), these data suggest that the GAGsi region responsible for gene silencing generates gene body-specific nucleosome positioning histone trimethylations (H3K36me3 and H3K79me3).

## IR creates a chromatin boundary

**Figure 4:**
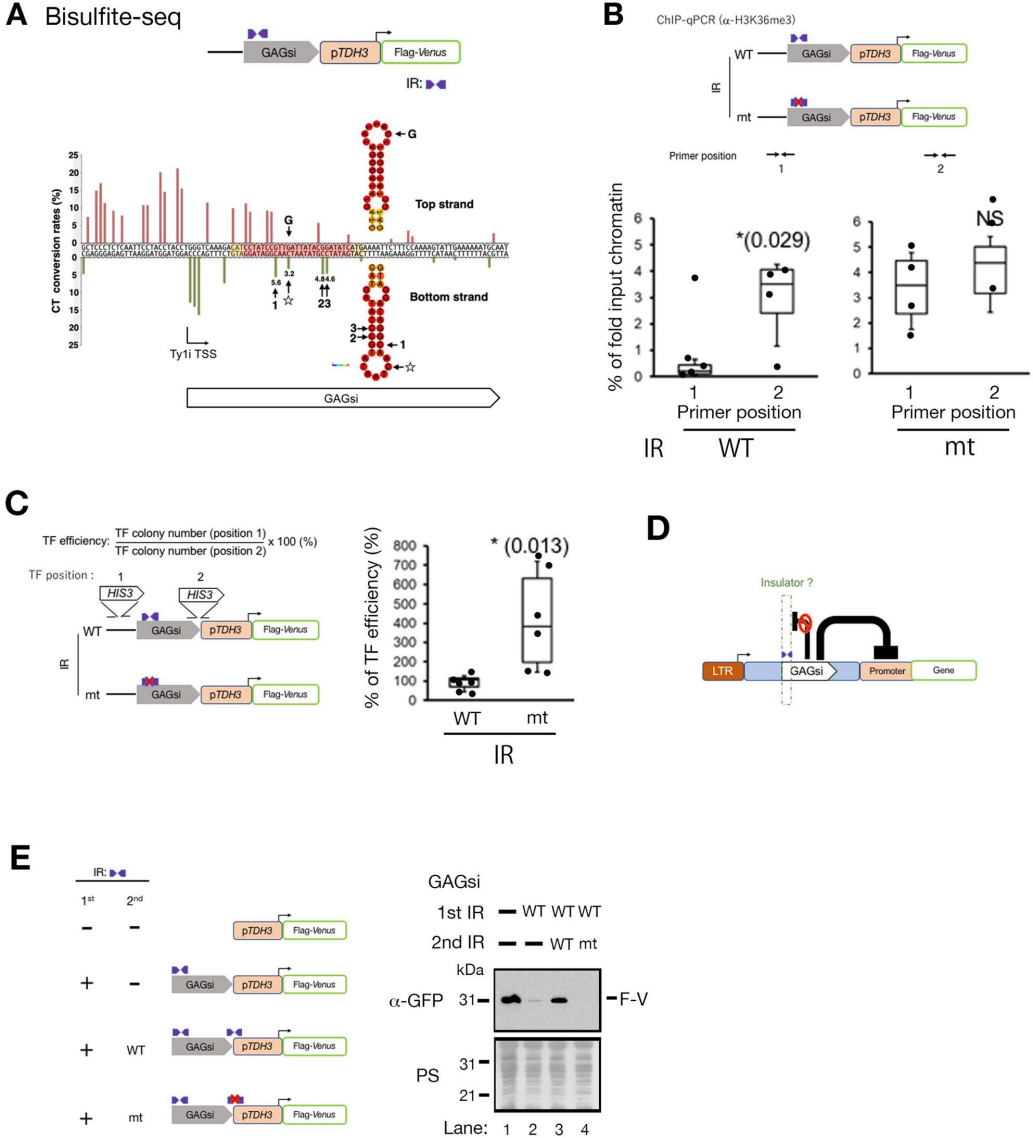
The IR in GAGsi functions as an insulator. (**A**) Bisulfite-sequencing (BS-seq) analysis of GAGsi sequence. The red and yellow sequences correspond to the IR region sequences. G: G on the loop region of IR (top strand). ☆: C on loop region of IR (bottom strand). The numbers (1, 2 and 3) on the plain sequence of bottom strand indicate the sequence position on the IR structure. The number on each sequence position at the IR structure (bottom strand) indicates the CT conversion rates (%). (**B**) ChIP-qPCR analysis using anti-H3K36me3. The positions of two primer sets used in this study (upper scheme). The results are the average ± sd (n ≤ 5, independent experiments) and analyzed by unpaired *t*-test (one-tailed). (**C**) Comparison of the transformation (TF) efficiency between WT IR-GAGSi and mt IR-GAGsi. The stem sequence mutant was used as mt IR-GAGsi. The positions of two *HIS3* gene integration sites used in this study, and the formula for calculating the TF efficiency (%) (upper scheme). The results are the average ± sd (n ≥ 5, independent experiments) and analyzed by unpaired *t*-test (one-tailed). The raw data is listed in Table S3C. (**D**) Schematic of IR sequence as an insulator. **(E**) The IR sequence functions as an insulator. An additional GAGsi IR sequence (either wild-type or stem-mutant) was inserted between the GAGsi and *TDH3* promoters as the 2nd IR (left cartoon). Immunoblot using an anti-GFP antibody to detect the expression of Flag-Venus (right).

The IR sequences were located at chromatin boundaries where histones H3K36me3 and H3K79me3 appear (Fig. 3C-E). We examined whether the IR actively forms a chromatin boundary separating the promoter-specific chromatin from the gene body chromatin (Figs. 4A and S4, Table S2). Chromatin relaxation can lead to exposure of naked DNA and even transient exposure of ssDNA. Bisulfite sequencing (BS-seq) converts unmethylated cytosines (C) to thymines (T) on single-stranded DNA (ssDNA) on a chromosome, and subsequent analysis steps focus on counting the number of C to T conversions and qualifying the methylation ratio per base, i.e. the frequency of ssDNA exposure on chromosome^25^. BS-seq was used to detect ssDNA exposure associated with gene transcription on chromatin in *Bacillus subtilis* and human B cells^26,27^. We used BS-seq to measure the frequency of ssDNA exposure near IR sites and to examine changes in chromatin structure around IR sequences. We used the GAGsi region without the 5'-LTR to eliminate the effect of transcription fork migration through the 5'-LTR (Fig. 4A: cartoon). The CT conversion rates centered on the IR site were high in the region upstream of the IR site, while the CT conversion rates downstream of the IR site remained low and gradually increased (Fig. S4 and Table S2). This suggests that the chromatin state changes around the IR site. We further investigated the change in CT conversion rates around the IR site. Figure 4A shows that on both the upper and lower strands upstream of the IR sequence (colored red), the CT conversion ratio reached 20%. However, the CT conversion ratio in the GAGsi region dropped to less than 5% with the IR sequence as the turning point. The IR sequences were able to form hairpin structures in vitro (Fig. 2D). However, the CT conversion of guanine (G: arrowhead in the hairpin structure (Fig. 4A)), which is present in the bubble sites and could expose the ssDNAs in the hairpin structure, did not increase significantly. This does not support that the IR sequence of GAGsi forms a hairpin structure in the chromosome. Thus, the IR sequence is the tipping point to change the state of DNA exposure in the chromatin. The IR creates a chromatin boundary, serving as a starting point for gene body chromatin modification with histone H3K36me and H3K79me3 (Fig. 3C-E).

Next, we investigated whether mutations in the IR sequence could disrupt chromatin boundaries. The histone H3K36me3 was present in the chromatin downstream of the IR together with the histone H3K79me3 (Fig. 3C-E). To test whether H3K36me3 extended upstream of the IR mutant sequence, we performed chromatin immunoprecipitation-quantitative PCR (ChIP-qPCR) for H3K36me3 and compared the levels of H3K36me3 between upstream and downstream of the IR site. To eliminate the impact of transcriptional fork migration from 5’-LTR, we utilized the GAGsi region without 5’-LTR (Fig. 4B: cartoon). We set the primer sets at the two positions upstream and downstream of IR, respectively (Fig. 4B: cartoon, primer positions). The amount of H3K36me3 at primer position 1 was significantly lower than that at position 2 in the IR WT, although the amount of H3K36me3 was almost the same between primer positions 1 and 2 in the IR mt sequence (Fig. 4B). These data suggest that the mutation of the IR sequence lost a chromatin boundary and extended histone H3K36me3-modified chromatin beyond the IR. We then investigated whether the IR mutant altered chromatin status. Efficient integration of DNA fragments via homologous recombination (HR) is indirectly affected by chromatin status. In budding yeast, transformation efficiency increases inversely with high nucleosome density in the gene body region, although the relationship between transformation efficiency and nucleosome density is not always correlated in a fuzzy chromatin structure of the actively transcribed gene region^28^. We calculated the transformation (TF) efficiency by the number of transformants integrating the *HIS3* marker gene at two target positions across the IR (Fig. 4C: cartoon). The TF efficiencies of the IR mutant were significantly higher than those of the IR WT (Fig. 4C). Thus, chromatin status upstream of the IR site can be altered by the IR mutant. Taken together, the IR sequence acts as a chromatin boundary, altering the chromatin state.

## The GAGsi IR functions as an insulator

A chromatin boundary has originally been found to insulate neighboring genes by blocking the influence of transcriptional enhancers or the spread of silent chromatin^29^. We investigated whether the IR site acts as an insulator against GAGsi silencing effect. (Fig. 4D). The DNA fragment (0.1 kb) containing an IR sequence that did not show a silencing effect was used as a secondary IR sequence (Fig. 2F). We tested whether the secondary IR sequence blocked the silencing effect of GAGsi by inserting it between GAGsi and the *TDH3* promoter (Fig. 4E). GAGsi repressed Flag-Venus expression in the absence of a secondary IR sequence (Fig. 4E: lanes 1 and 2). However, Flag-*Venus* was expressed under the GAGsi domain with the wild-type secondary IR sequence, but not with the mutant secondary IR sequence (Fig. 4E: lanes 3 and 4). Thus, to direct the GAGsi silencing effect downstream, the IR sequence acts as a chromatin insulator.

## DNA repair proteins, Esc2 and Rad57, participate in GAGsi silencing

**Figure 5:**
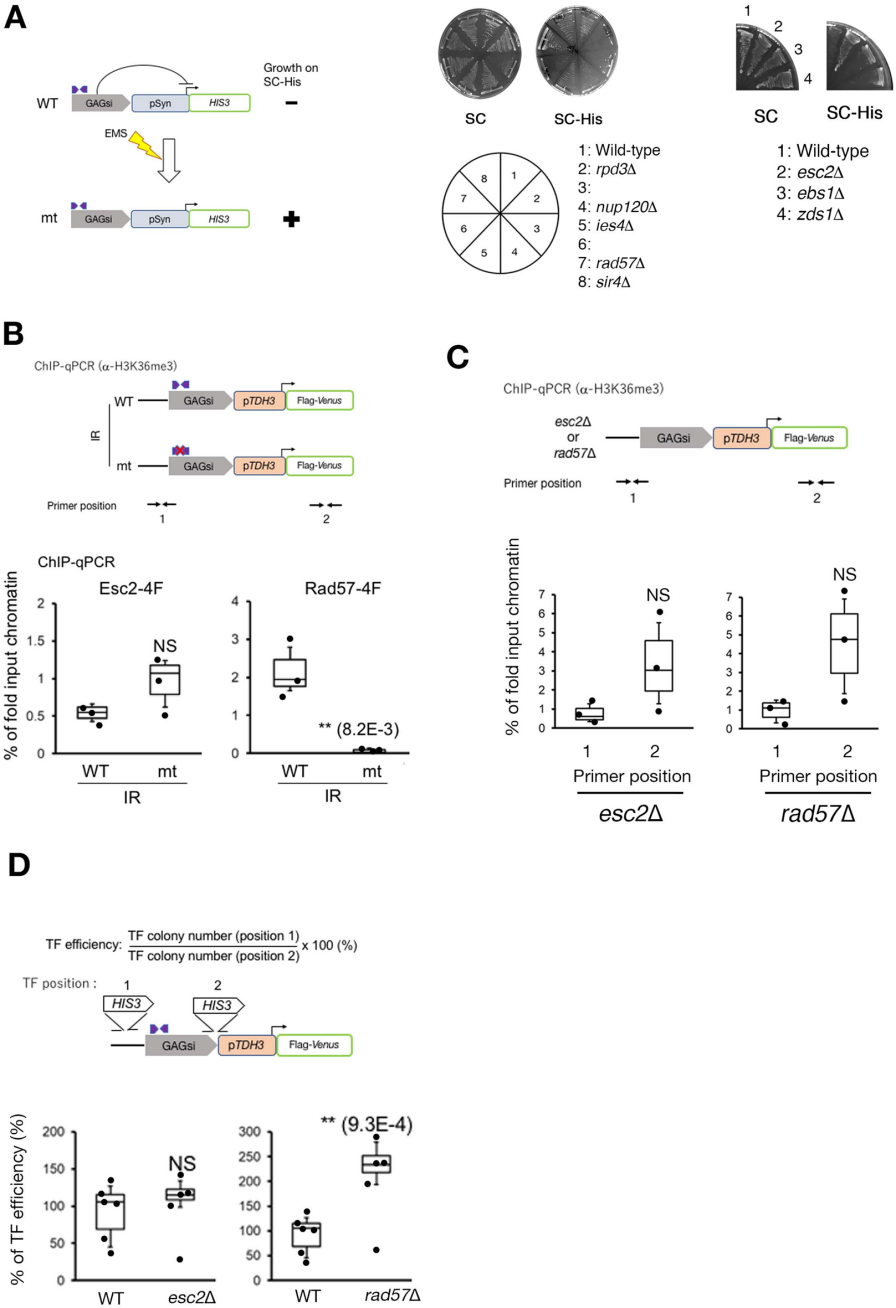
Esc2 and Rad57 regulate the silencing effect of GAGsi. (**A**) Schematic of gene screening to control GAGsi silencing (left cartoon). The plate assay to monitor cell growth of gene deletion strains in SC or SC-His plate (right). Each deletion strain harbored the GAGsi region upstream of the *HIS3* gene under the pSyn promoter. (**B**) ChIP-qPCR to monitor the localization of Esc2-4F and Rad57-4F in the GAGsi region. The positions of the primer sets are shown in the upper panel. The results, analyzed by unpaired t-test (one-tailed), are the average ± sd of three independent experiments (n = 3). (**C**) ChIP-qPCR analysis using anti-H3K36me3 antibody. The positions of the primer sets are shown in the upper panel. The results, analyzed by unpaired t-test (one-tailed), are the average ± sd of three independent experiments (n = 3). (**D**) Comparison of the TF efficiency between WT IR-GAGSi and mt IR-GAGsi. The positions of two *HIS3* gene integration sites used in this study (upper scheme). The results are the average ± sd (n ≥ 5, independent experiments) and analyzed by unpaired *t*-test (one-tailed). The raw data is listed in Table S3C.

To investigate which gene products are necessary for GAGsi silencing, we screened for a gene that, when mutated or deleted, eliminated GAGsi silencing. We used the parent strain HMY1605, which contains the *HIS3* gene under the pSyn promoter adjacent to the GAGsi region (Fig. 5A: cartoon). As the pSyn promoter is moderately active^30^, the GAGsi region effectively silenced the expression of the *HIS3* gene, and HMY1605 was unable to grow in the synthetic complete medium depleted of histidine (SC-His) plates (Fig. S5A: WT). We treated the HMY1605 strain with ethyl methanesulfonate (EMS) as a DNA mutagen to introduce genomic DNA mutations^31^, and then isolated four mutant strains that could grow on SC-His agar plates (Fig. S5A: mt1 ~mt4). We attempted to identify the causative mutations by complementation, transforming four different HMY1605 EMS-treated his + strains with a genomic library, but were unable to identify the responsible gene. As another approach, we examined the whole genome sequences of four EMS-treated and parent HMY1605 strains and identified the genes with novel mutations in each EMS-treated strain compared to the parent HMY1605 strain (Table S3A). We classified the isolated genes with novel mutations into each cellular function group using the Gene Ontology (GO) Term Finder in the *Saccharomyces* Genome Database (SGD) (https://www.yeastgenome.org). In this study, we focused on genes involved in DNA metabolism that directly affect GAGsi silencing (Table S3B), and selected genes among them whose deletion abolished the GAGsi silencing effect. HMY1605 strains lacking *RAD57* or *ESC2* genes grew on SC-His plates (Fig. 5A: plate data). Rad57 is a member of the Rad52 epistasis group and functions in recombinational repair of DNA double-strand breaks (DSBs)^32,33^. The cells lacking a gene in the Rad52 epistasis group, including *RAD51, RAD52*, *RAD54,* and *RAD55,* did not grow on the SC-His plate (Fig. S5B), suggesting that the role of Rad57 in GAGsi silencing is not related to the functions performed by the Rad52 epistasis group. Esc2 plays a role in several DNA metabolism pathways, including replication-associated recombination repair, gene silencing at HM loci, chromatin cohesion, and intra-S phase checkpoint^34–39^. Esc2 interacts with Srs2 and Elg1 to promote recombination at sites where replication forks are stalled^40^. Strains lacking the *SRS2* or *ELG1* genes did not grow on the SC-His plate (Fig. S5B), suggesting that the function of Esc2 in GAGsi silencing is also independent of its known functions with Esc2 binding partners. Next, we investigated whether Esc2 and Rad57 were able to bind to the GAGsi domain and whether this binding was IR-dependent. We carried out ChIP-qPCR for 4-Flag (four tandemly linked Flag epitope tag units) bound to Esc2 (Esc2-4F) or Rad57 (Rad57-4F) using an anti-Flag antibody. Esc2-4F was bound to the GAGsi domain with both WT IR and mt IR sequences (Fig. 5B: Esc2-4F). In contrast, Rad57-4F was bound to the GAGsi domain with the WT IR sequence, but not to the mt IR sequence (Fig. 5B: Rad57-4F). Next, we investigated the localization of both factors at Ty1 elements in the genome using ChIP-seq data for Esc2-4F and Rad57-4F. We used the chromatin immunoprecipitation-sequencing (ChIP-seq) method to investigate the presence of Esc2 and Rad57 on Ty1 elements registered in the SGD database. We calculated the average read counts at 31 different Ty1 elements for the IP fractions of Esc2-4F and Rad57-4F. As shown in Fig. S6, Esc2-4F and Rad57-4F did not bind from the 5' LTR region to the IR site of GAGsi, but rather in the region downstream of the IR site. Thus, Esc2-4F and Rad57-4F bind to gene-body chromatin formed downstream of the IR site on the Ty1 element. We then examined whether Esc2 or Rad57 affected the chromatin boundary between the promoter-specific chromatin and gene-body chromatin at the GAGsi domain using ChIP-qPCR with an anti-H3K36me3 antibody. The H3K36me3 level at primer position 1 was consistently lower than the amount at position 2 in both the *esc2*Δ and *rad57*Δ strains (Fig. 5C). However, statistical analysis indicated that there was no significant difference compared to the wild-type strain (Fig. 4B: IR WT). This suggests that the deletion of *esc2* or *rad57* may have extended the gene body chromatin with H3K36me3 beyond the IR site and into upstream regions. Furthermore, the TF efficiency to integrate the *HIS3* gene fragment at positions 1 and 2 across the IR sequence of GAGsi was almost the same between WT and *esc2*Δ (Fig. 5D: left graph), although the TF efficiency was significantly increased in *rad57*Δ more than in WT (Fig. 5D: right graph). This suggests that deletion of *rad57* results in the alteration of chromatin structure, similar to the case of mt IR sequence (Fig. 4B), whereas deletion of *esc2* does not.

## GAGsi supports Ty1 transcription from the 5’-LTR

**Figure 6:**
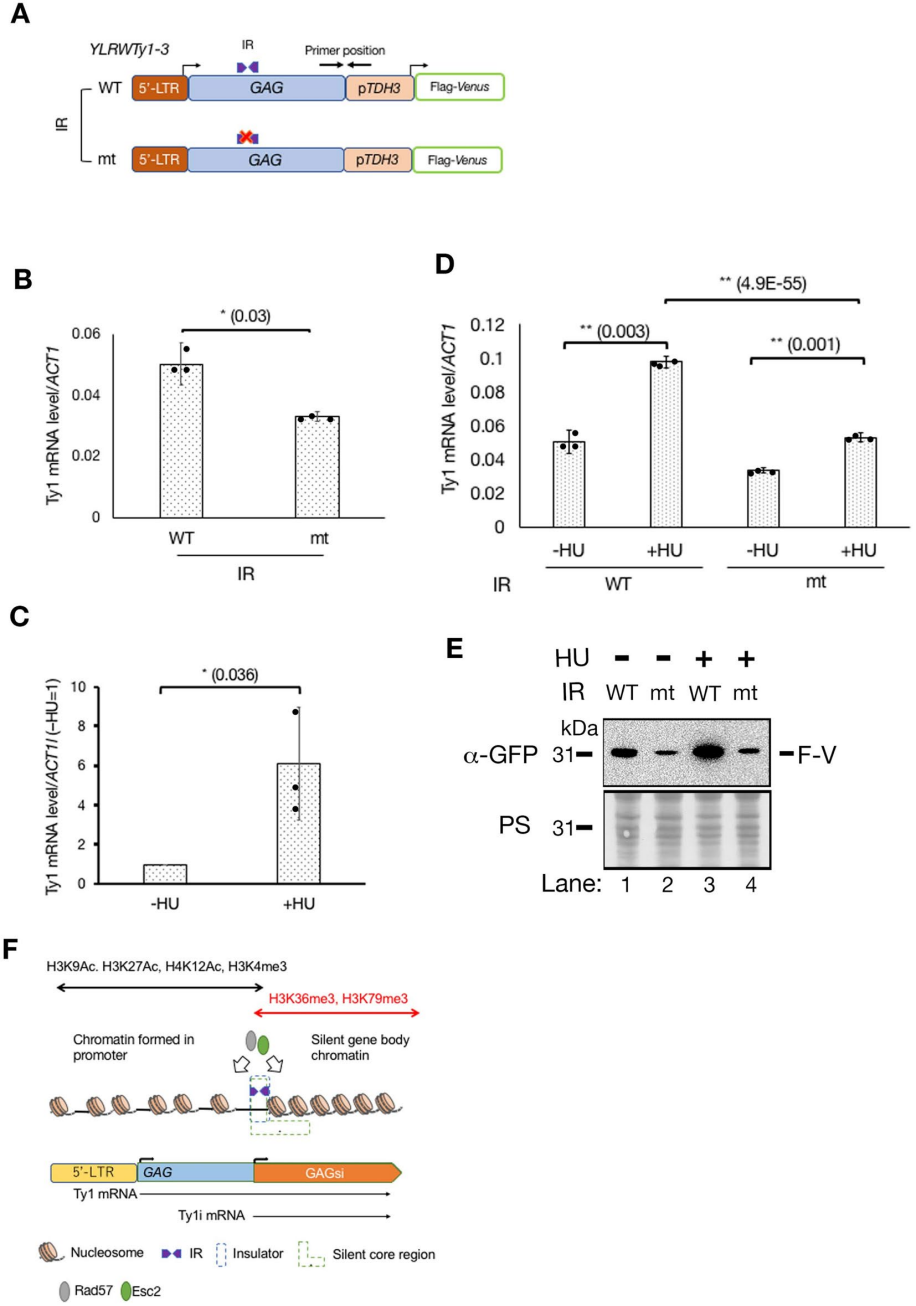
GAGsi assists Ty1 transcription from the 5’-LTR. (**A**) The region from the 5’-LTR to the 3' terminus of the *GAG* gene in *YLRWTy1-3* was connected to p*TDH3*-Flag-Venus. The primers between GAGsi and p*TDH3* were used for RT-PCR. **(B**) RT-PCR analysis for Ty1 mRNA levels in the WT IR-GAGSi and the mt IR-GAGsi. (**C**) RT-PCR analysis for Ty1 mRNA levels of the WT IR-GAGsi in GAGsi with or without HU treatment. (**D**) RT-PCR analysis for Ty1 mRNA levels either in the WT IR-GAGSi and the mt IR-GAGsi with or without HU. The Ty1 mRNA data of the WT IR sequence and the mt IR without the HU (-HU) are the same as the Ty1 mRNA data in the WT and in the mt in (**B**). The results were average ± s. d. (n = 3, independent experiments) and analyzed by unpaired *t*-test (two-tailed). **(E**) Immunoblot comparing the expression levels of Flag-Venus (F-V) in either WT-IR or IR-mt in 5’-LTR + *GAG* with or without HU treatment. Anti-GFP antibody was used in the immunoblot. (**F**) The chromatin structure surrounding GAGsi region.

Since the transcription factor binding sites are widely distributed from the 5' LTR to the TSS of Ty1i (Fig. 1A)^3^, the promoter-like chromatin formed by GAGsi is required for the transcription of Ty1 itself and the induction of Ty1 itself in response to cell stress. We therefore quantitatively compared whether Ty1 transcription is affected by GAGsi and its effect on the induction of Ty1 transcription under cellular stress. To distinguish the mRNA species of the target Ty1 from the 5’-LTR from other Ty1 elements, we combined the sequence from the 5’-LTR to the *GAG* gene sequence of *YLRWTy1-3* with the *TDH3* promoter, and monitored the mRNA level using primers to amplify the junction between the *GAG* gene and the *TDH3* promoter (Fig. 6A). We used RT-PCR to compare the level of transcription of this Ty1 fusion between WT and mt IR sequences. The mRNA level of the mt IR sequence in the GAGsi domain was significantly reduced compared to the WT IR sequence (Fig. 6B). We then investigated whether the GAGsi IR mutation affected the induction of Ty1 transcription by cellular stress. Several cellular stresses, such as DNA damage and nutrient deprivation, induce Ty1 transcription^3^. Hydroxyurea (HU) is an inhibitor of ribonucleotide reductase and this treatment leads to DNA replication fork stalling as cellular stress^41,42^. We confirmed that HU treatment was able to induce Ty1 mRNA levels (Fig. 6C). Ty1 mRNA increased more with HU treatment than without HU treatment in both WT and mutant IR sequences (Fig. 6D: + /- HU). However, the Ty1 mRNA level in the WT IR sequence was significantly higher than in the IR mutant in the presence of HU (Fig. 6D: WT (+ HU) vs. mt (+ HU)). Thus, GAGsi supports Ty1 transcription from the 5’-LTR by creating promoter-specific chromatin upstream of the IR site.

The construct used in this study contains transcriptional regulation units for Ty1 and Ty1i transcriptions in its 5’-LTR -GAG gene (Fig. 6A). We tested whether mutation of the IR sequence in GAGsi and addition of HU would affect Ty1 or Ty1i transcriptional elongation. RNA extracted from the cells was converted to DNA by reverse transcription, and the size of each mRNA was examined by PCR using a combination of five primer sets (Fig. S7: cartoon). The mRNA size in WT IR without HU was consistent with the size from the 5' LTR to the 3' end of Flag-Venus (Fig. S7: Primer position A: lane 2). However, HU treatment prevented amplification of DNA of this size (Fig. S7: Primer position A: lanes 2 and 3). In the case of IR-mt, HU treatment slightly decreased the amount of mRNA (Fig. S7: Primer position A: lanes 4 and 5). In this study, we used the primers to detect both Ty1 mRNA and Ty1i mRNA placed downstream of IR (Fig. S7: cartoon, Primer positions B and D). If Ty1i mRNA was induced in response to HU treatment, we expected that PCR pattern should be altered, but HU treatment reduced the mRNA levels in IR-WT and IR-mt similar to the mRNA elongation pattern from the 5'-LTR (Fig. S7: Primer positions A and B). This suggests that Ty1i mRNA is either not present sufficiently to be significantly detected by PCR or is present but like Ty1 mRNA, elongation arrests upon HU treatment. We hypothesized that Ty1 mRNA does not elongate to the 3' end of Flag-Venus upon the addition of HU because the transcription fork arrests in the vicinity of the *TDH3* promoter following activation of the *TDH3* promoter by HU treatment. To test this hypothesis, we examined mRNA elongation from the 5' LTR or downstream of the IR to the *TDH3* promoter, and found no change in the amount of PCR product synthesized with or without HU or mutation to the IR (Fig. S7: Primer positions C and D). The amount of Flag-Venus transcription also remained unchanged under each condition (Fig. S7: Primer position E). Thus, the transcription fork originating from the 5'-LTR stops near the *TDH3* promoter induced by HU treatment, and a new transcription fork originating from the *TDH3* promoter passes through the Flag-Venus region.

The genomic Ty1 elements contain 5’-LTR that acts as a promoter upstream of the GAGsi domain. We investigated the effect of this 5’-LTR on the silencing effect of GAGsi. When the IR sequence was WT, Flag-Venus expression occurred even in the absence of HU. In the presence of HU, Flag-Venus expression increased (Fig. 6E, lanes 1 and 3). This is likely because the silencing effect of GAGsi is counterbalanced by the passage of the transcription fork as a result of frequent transcription of Ty1, and transcriptional activation from the 5’-LTR by the addition of HU further attenuated the silencing effect of GAGsi. Unexpectedly, Flag-Venus transcription was reduced in IR-mt compared to WT (Fig. 6E, lanes 1 and 2), and HU addition did not increase F-V expression (Fig. 6E, lanes 2 and 4). This suggests that the chromatin conformational changes caused by the IR mutation not only reduce the amount of transcription forking from the 5'-LTR, but also repress downstream Flag-Venus gene expression.

## Discussion

In this study, we identified GAGsi as a silencing region within the *GAG* gene in the Ty1 element (Fig. 6F). The IR sequence in the GAGsi region is not only necessary for the silencing effect to occur but also acts as an insulator to restrict the silencing effect in the downstream direction. The IR sequences exist as chromatin boundaries separating the promoter-specific chromatin from the gene-body chromatin. Euchromatin and heterochromatin each harbor a unique histone modification pattern, and gene silencing is closely correlated with heterochromatin structure^43^. In contrast to the heterochromatin-mediated gene silencing mechanism found in budding yeast^44,45^, GAGsi silent chromatin is a typical gene body chromatin structure characteristic of euchromatin. Thus, gene silencing does not always require the heterochromatin structure and its gene repression effect can also occur in chromatin with euchromatin-specific histone modification patterns. The IR of GAGsi not only contributes to the silencing function, but also serves as a chromatin boundary. The IR sequence is located in the 5' UTR of Ty1i, and it has been reported that the IR sequence forms a hairpin structure (hairpin 1 (H1)) within free Ty1i RNA molecules^18^. The BS-Seq data do not support that IR forms hairpin structures in vivo (Fig. 4A). However, the dramatic change in ssDNA exposure on chromatin before and after IR suggests the presence of a factor that recognizes and binds to IRs. Additional screening for elements that interact with the IR is warranted. An unidentified factor may hold significant importance in the silencing impact of GAGsi and in the formation of chromatin boundaries by the IR.

We isolated Esc2 and Rad57 as factors responsible for the GAGsi silencing effect. ChIP-qPCR results showed that Rad57 binds to the GAGsi region, with binding dependent on the IR sequences, and Esc2 could bind to the GAGsi region regardless of the mutation of the IR sequence (Fig. 5B). Furthermore, ChIP-qPCR data using antibodies against histone H3K36me3 showed that deletion of *esc2* and *rad57* does not significantly affect the structure of the promoter-specific chromatins and gene body chromatin formed in the GAGsi region (Fig. 5C). These data suggest that Esc2 is involved as a component of higher-order silent chromatin by binding to gene-body chromatin in the GAGsi region. Given that the binding of Rad57 to GAGsi is IR-dependent and that Rad57, like Esc2, is localized throughout Ty1 gene region (Fig. S6), we speculate that the binding of Rad57 to the vicinity of the IR in GAGsi is dependent on a mechanism that forms chromatin boundaries around the IR. It is speculated that the binding of Rad57 to the vicinity of the IR in GAGsi is dependent on the mechanism of chromatin boundary formation centered on the IR. Furthermore, the removal of either *esc2* or *rad57* gene led to a substantial decline in Ty1 transcription (Fig. S8). It is conceivable that the localization of these two factors, in conjunction with a group of other transcription factors, is necessary for the activation of Ty1 transcription itself.

Insulators or boundary elements are genetic elements located near chromatin domain boundaries that have distinct properties involved in changes in gene expression^46^. They act as a barrier to prevent the spread of adjacent repressive heterochromatin domains with specific histone modifications, such as the 5'-HS4 insulator element of chicken ß-globulin^47^. In eukaryotes, it has been reported that IRs function as chromatin boundaries that separate euchromatin from heterochromatin^48–50^. IR sequences not only exert their silencing effect as part of GAGsi, but also unidirectionally limit the silencing effect of GAGsi through their insulator function. In addition, the IR sequence prevents the penetration of transcriptionally repressive gene-body chromatin with histones H3K36me3 and H3K79me3 beyond the GAGsi region into the TSS region of Ty1. This insulator/chromatin boundary function of IR allows GAGsi to promote transcription of the Ty1 element itself by creating an promoter-specific chromatin region from the 5’-LTR to the IR sequence, facilitating the binding of a group of transcription factors to their target sequences. This heterochromatin extends bidirectionally from the silencing core sequence across the retrotransposon sequence to the surrounding gene cluster^51,52^. Because the gene clusters comprising the Ty1 genome are encoded by a single mRNA from the 5’-LTR, GAGsi silencing does not affect the expression of genes in the Ty1 genome. In addition, the silencing effect of GAGsi is also short (~ 1 kb), so it is unlikely that GAGsi silencing affects a set of genes outside of the Ty1 locus. However, it is possible that an IR-centered silencing mechanism similar to GAGsi could affect the expression of a group of non-retrotransposon genes. In humans, the IR-structured gL region derived from the non-LTR retrotransposon Line-1 represses the expression of downstream HLA genes^19^. IR-centered silencing mechanisms may be involved in the transcriptional repression of genes in a variety of species.

## Methods

### Growth, media, yeast strains, and transformation

*E. coli* strain DH5*α* was used for plasmid transformation. *E. coli* cells were routinely cultivated in standard LB medium (1% bactotryptone (catalog no. 211705; BD), 0.5% yeast extract, 0.5% NaCl) with the addition of appropriate antibiotics (ampicillin, 100 µg/ml; kanamycin, 50 µg/ml). All plasmids were introduced into DH5*α* competent cells according to Hanahan’s method^53^.

Table S1 lists the genotypes of the budding yeast strains used in this study. The parental budding yeast strain used in this study was BY4742 (MATα *his3 leu2∆1 met15∆0 ura3∆0*)^54^. A yeast strain with a single gene deletion was commercially available from the haploid yeast open reading frame deletion collection (Horizon Discovery, Cambridge, UK). Yeast cells were grown routinely at 30 °C in yeast extract-peptone-dextrose (YPD) (1% yeast extract, 2% bacto-peptone (catalog number 211677; Thermo Fisher Scientific, Waltham, MA, USA) and 2% glucose) or appropriate synthetic complete (SC) medium supplemented with amino acids^55^. Media were solidified by adding 2% agar when necessary. For time course analysis and bisulfite sequencing, yeast cells were cultured at 25 °C. A standard method was used to isolate yeast genomic DNA. This method has been described elsewhere^55^.

To construct yeast strains harboring different types of GAGsi regions, we used the HMY1462 strain as the base strain (Table S1). By integrating the PHM761 plasmid into the *TRP1* locus of the BY4742 strain, this strain harbors the *ura3-1* mutant gene at the *TRP1* locus (Fig. S9). The *URA3* plasmid carrying different GAGsi constructs was treated with *Nco* I restriction enzyme to cut at the *URA3* gene. The *Nco* I digested *URA3* plasmid was transformed into the HMY1462 strain and the plasmid integrated into the *ura3-1* locus to obtain the *URA*+ transformants.

Yeast transformation has been described elsewhere^56^. Yeast cells were grown on YPD solid plates, collected with toothpicks, and suspended in 100 µL of one-step buffer (0.2 M lithium acetate (LiAc), 40% polyethylene glycol (PEG), 100 mM dithiothreitol (DTT)) containing a plasmid or linearized plasmid mixture. The mixture containing 0.5–1.0 µg of integration plasmid (*LEU2* or *HIS3* marker gene) was used for transformation. The cell suspension was incubated at 42 °C for 1 h and then plated on SC agar medium depleting uracil or other amino acids, if necessary. Correct integration of the plasmid into the resulting colony was confirmed by PCR.

### PCR amplification of DNA fragments for plasmid construction

PCRs for plasmid construction were performed using iProof high-fidelity DNA polymerase (catalog no. 1725301; Bio-Rad, Hercules, CA, USA). Oligonucleotides were supplied by FASMAC (Kanagawa, Japan), and restriction endonucleases, and T4 DNA ligase were obtained from New England BioLabs (Ipswitch, MA, USA). All relevant plasmid fragments were verified by DNA sequencing.

DNA fragments were amplified from 10 ng template DNA in a 50 µL PCR reaction (10 µL 5× iProof reaction buffer, 200 μM dNTPs, 0.5 µM forward and reverse oligonucleotides as primers, and 1 U iProof high-fidelity DNA polymerase) using the following PCR program 30 s at 98 °C, 30× (10 s at 98 °C, 15 s at 53 °C, 60 s at 72 °C) and 5 min at 72 °C. After purification of the PCR products using the Wizard SV Gel and PCR Clean-Up System (catalog number A9281; Promega, Madison, WI, USA), the DNA amplified fragments were digested with restriction enzymes for ligation.

### Analysis of DNA secondary structure: Mung-Bean nuclease (MBN) treatment and PCR analysis

To detect the DNA secondary structure on a plasmid, 200 ng of plasmid (PHM888 and PHM974) was incubated at 30 °C for 10 min with or without 10 units of MBN (catalog no. B025S: New England BioLab) (final volume 10 μL). To quench the MBN reaction, 5 μL of the reaction was mixed with ethylenediaminetetraacetic acid (EDTA) at a final concentration of 1 mM and incubated at 65 °C for 5 min. The reaction quenched sample was mixed with 70 μL of DIW and stood on ice until use.

The PCR mixture was prepared with 1 μL of the reaction-quenched sample (1.25 ng of DNA), 5 µL of enzyme mixture of the PCR reaction (AmpliTaq Gold 360 master mix, catalog no. 10289234: Fisher Scientific, Waltham, MA, USA) and primers at a final concentration of 2 μM each (final volume 10 μL). The primers used in this reaction are listed in Table S1. Reactions were run for 1 cycle of 10 min at 95 °C; 30 cycles of 30 s at 94 °C, 30 s at 53 °C, and 30 s at 68 °C; 1 cycle of 7 min at 68 °C. The PCR reaction was run on a 1.5% agarose gel and electrophoresed at 100 V. After staining the agarose gel with ethidium bromide, the DNA bands were visualized by ultraviolet (UV) irradiation.

### Galactose-induced protein expression

As a preculture, the HMY1559 strain was inoculated into 2 mL of YPR (1% yeast extract, 2% bacto-peptone (catalog no. 211677; Thermo Fisher Scientific, Waltham, MA, USA), 2% raffinose) and cultured overnight at 25 °C. Cultured cells were suspended in 5 mL of YPR at OD600 = 0.5 and mixed with either galactose or glucose to a final concentration of 2%. Cells were cultured at 25 °C for 3 h. After the cells were harvested, whole cell extracts were prepared as described previously^57^. Target protein expression was analyzed by immunoblotting.

### Bisulfite sequencing (BS-seq)

Log-phase HMY1613 cells (OD_600_ =  ~ 0.9) were grown in YPD medium at 25 °C with shaking, and 1 ml of the culture was used for the sodium bisulfite treatment. After harvesting, the cell pellet was resuspended in 500 μL of an RNA stabilizing reagent (RNA*later* Solution, catalog no. AM7020: Thermo Fisher Scientific, Waltham, MA, USA) and incubated at room temperature for 10 min. After harvesting, the cell pellet was quickly frozen in liquid nitrogen and stored at − 80 °C until use. Aqueous 80% methanol (methanol: water, 80:20 v/v) was directly added to the frozen cell pellet to dehydrate and immobilize proteins, DNA, and RNA and to permeabilize cell membranes to get sodium bisulfite to be easier to enter into the cell and left for 5 min, and then the cells were collected by centrifugation. After discarding the supernatant, the cells were lysed with Zymolase 100 T (catalog no. 07665-55: Nacalai Tesque, Kyoto, Japan) at concentrations of 2.5 mg/ml in a buffer (100 mM Phosphate buffer, pH 7.5, 1.5 M Sorbitol) for 30 min at 37 °C. Sodium bisulfite treatment was carried out by using both EpiTect Fast Bisulfite Kit (catalog no. 59802: Qiagen (Germantown, MD, USA) and EpiTect Fast LyseAll Bisulfite Kit (catalog no. 59864: Qiagen) according to the manufacturer’s instructions with several; the spheroplast cells were directly treated with a mixture of sodium bisulfite and DNA protect buffer before Proteinase-K treatment and cell lysis to analyse CT conversion with nucleosome structure to be maintained. We carried out the sodium bisulfite reaction at 45 °C for 180 min without DNA denaturation, then cells were treated with Proteinase-K and Lysis Buffer FTB. After the bisulfite-treated DNA was eluted from the DNA spin column, the DNA was precipitated with ethanol, rinsed with 70% ethanol, resuspended with Milli-Q water, and stored at -80 °C until use. To amplify the Ty1 region for sequencing, the target region was divided into two parts. The two DNA fragments were amplified by PCR (TaKaRa EpiTaq HS, catalog no. R110A: TAKARA, Kusatsu, Japan) using the following primer sets: for the first part, YLRWTy1-3-upF1.1/YLRWTy1-3-inR0.1 and for the second part, YLRWTy1-3-upF4/YLRWTy1-3-inR1.4. The primer sequences are listed in Table S1. The PCR fragment was purified by precipitation with PEG 8000 as follows; equal volumes to the PCR reaction of PEG solution (20% (w/v) PEG 8000, 2.5 M NaCl) were added and centrifuged. After rinsing with 70% ethanol and drying up, the DNA fragment was resuspended in Milli-Q water. The DNA fragments' concentration was quantified using a fluorescence dye and a fluorometer (Qubit dsDNA BR assay kit, catalog no. Q32850: Thermo Fisher Scientific). The amplified DNA fragments (100 ng) were sheared to the average length of 250 bp by using an ultrasonicator (Covaris focused-ultrasonicator S220: Covaris, Woburn, MA, USA) and purified by using magnetic beads (Agencourt AMPure XP, catalog no. BC-A63880: Beckman Coulter, Brea, CA, USA). Adaptor and index primers were added to the sheared DNA fragments using a next-generation sequencing library preparation kit (NEBNext Ultra DNA Library Prep Kit for Illumina (Brea, CA, USA), catalog no. E7370S: NEB) according to the manufacturer’s instructions. PCR enrichment was carried out with nine cycles. The index-ligated fragments were mixed to construct a sequencing library and sequenced using the MiSeq system and its reagent kit (MiSeq Reagent Kits v2 (500 cycles), catalog no. MS-102-2003: Illumina) according to the manufacturer’s instructions. Sequencing was carried out with 250 cycles and a paired-end mode. Sequenced reads were trimmed using a trimming tool, Trimmomatic (v0.33)^58^ with the following parameters: accepted mismatches, 2; palindrome clip threshold, 30; simple clip threshold, 10; leading, 20; trailing, 20; window size, 4; average quality, 15; minimal length, 30. The trimmed reads were mapped to the reference sequence by using the mapping software, Bismark (v0.20.0)^59^. The version of Bowtie2, the software required to run Bismark, was v2.2.6^60^. The parameters for the minimum alignment function of Bismark (score-min option) used were L, -100, -2. Mapped reads below MAPQ <  = one were removed using SAMtools (v1.9)^61^, and reads with insertions and deletions were removed using an in-house Perl script pid3.pl (available at https://github.com/KoichiYanoNIG/pid3). Depths of the coverage for each nucleotide position in the mapped reads were counted for four bases (A, T, G, C) by using igvtools^62^. CT conversion rates were calculated for each cytosine on both the top and bottom strands according to the following equations: CT conversion rates for top strand (%) = (Counts for T) /(Counts for C + Counts for T) × 100; CT conversion rates for bottom strand (%) = (Counts for A) / (Counts for G + Counts for A) × 100. The CT conversion rates at the nucleotide positions from 23rd to 1919th and from 132nd to 2434th for the first and second part of the target region, respectively were used.

### Mutagenesis

The genetic screen utilized the HMY1605 strain as the parental strain. Mutagenesis using ethyl methanesulfonate (EMS) was described elsewhere^55^. Briefly, the HMY1605 strain was cultured overnight at 25 °C. Cells (2 × 10^8^ cells) were harvested and washed twice with distilled H2O (dH2O). The cells were suspended in 1 ml of 0.1 M sodium phosphate pH 7.0 and mixed with 30 µL of EMS (catalog no. 325-81522; Fujifilm, Tokyo, Japan). The cell suspension was incubated at 30 °C for 1 h and harvested. The cell pellet was washed three times with 200 μL 5% sodium thiosulfate and suspended in 1 mL dH2O. The EMS-treated and control cells were diluted in dH2O at 1 × 10^5^ cells/ml. A small aliquot of the cell suspension (1 × 10^4^ cells/aliquot) was plated on the SC-His plate (total 2.5 × 10^5^ cells/25 SC-His plates) and incubated at 25 °C for 3 days. The resulting *HIS*+ colonies (total 29 colonies) were streaked onto fresh SC-His plates and incubated for 4 days to eliminate the false positive colonies. Finally, 4 *HIS*+ colonies were obtained and stored until use.

### Whole-genome sequencing (WGS)

The HMY1605 parent strain and four HMY1605 mutant strains (HMY1605 EMS1/2/3/4) were used for WGS analysis to identify mutations. A standard method described elsewhere^55^ was used to isolate yeast genomic DNA. WGS libraries were prepared using the Collibri PS DNA Library Prep Kit according to the manufacturer's protocol (catalog no. A39122024; Invitrogen) and sequenced on an Illumina NovaSeq 6000 system using the 150 base pair pair-end sequencing kit. Sequence analysis was performed by Novogene (Beijing, China). The sequencing data (DRA012864) were uploaded to the Galaxy web platform (https://usegalaxy.org) for data analysis^63^. Variants were identified and annotated with the Snippy v.4. 6. 0^64^. We filtered out variants that were found in the HMY1605 parental strain that we sequenced, leaving us with novel mutations in protein-coding genes in the HMY1605 mutant strains (Table S3A). Of these mutations, mutations in genes categorized in DNA metabolism were retrieved using the gene ontology (GO) term finder of the *Saccharomyces* genome database (SGD) (https://www.yeastgenome.org).

### Immunoblot analysis

Immunoblotting was performed using antibodies against the flag epitope (catalog no. F3165; Sigma-Aldrich, Burlington, MA, USA) or GFP (donated by the Francis Crick Institute) at 1:1,000 dilution as the primary antibody. Whole-cell extracts were prepared as previously described^57^, separated on an SDS–polyacrylamide gel electrophoresis gel, and transferred to Amersham Protran 0.45 µM NC (catalog no. 10600002; GE Healthcare, Chicago, IL, USA) by wet electroblotting with sodium carbonate transfer buffer containing 20% methanol at 0.5 A for 1 h at 4 °C^65^. The membrane was washed with tris-buffered saline with Tween 20 (TBS-T; 20 mM Tris–HCl, pH 7.5, 150 mM NaCl, 0.1% Tween 20) and stained with 0.1% Ponceau S solution (catalog no. P3504; Sigma-Aldrich) to confirm the protein level in each sample. The blots were incubated with primary antibodies using 5% skim milk in TBS-T for 1 h at room temperature. After three washes with TBS-T, the blots were incubated with goat anti-mouse immunoglobulin G (IgG) (Fab specific) peroxidase antibody (Catalog No. A9917; Sigma-Aldrich) as a secondary antibody with 5% goat milk in TBS-T for 1 h at room temperature. The blots were then washed three times with TBS-T and developed using SuperSignal West Dura Stable Peroxide (catalog no. 1856146; Thermo Scientific). The band signal was analyzed using a ChemiDoc Touch MP imaging system (Bio-Rad). The amount of protein in each lane was adjusted using Ponceau S staining. This has the advantage of allowing the comparison of protein bands of different sizes compared to the loading control method using housekeeping proteins^66^.

### ChIP-sequencing (seq)

The ChIP sample preparation protocol was modified from a previously described method^67^. The strains used for ChIP-qPCR were HMY1613 and HMY1751 (Table S1). Log-phase yeast cells (OD600 = 0.5–0.6, 25 ml of culture volume) were crosslinked in formaldehyde at a final concentration of 1% in YPD medium (15 min at 25 °C) and then quenched with glycine at a final concentration of 125 mM for 5 min at 25 °C. The cells were harvested and washed twice with cold TBS buffer (20 mM Tris–Cl pH 7.5, 150 mM NaCl). Cells were resuspended in 400 µL ChIP NP-S buffer (0.5 mM spermidine, 1 mM ß-mercaptoethanol, 10 mM Tris–Cl pH 7.5, 50 mM NaCl, 5 mM MgCl2, 1 mM CaCl2, 0. 08% NP-40 and protease inhibitor cocktail (cOmplete (catalog no. 1697498001; Merck, Rahway, NJ, USA)) and mixed with the same volume of acid-washed glass beads (catalog no. G8772; Sigma-Aldrich). Cells were disrupted with the Mini bead beater (Biospec Products, Bartlesville, OK, USA) (30-s beating, 30-s cooling for 5 cycles) and the cell suspension was collected. Chromatin in the cell suspension was sonicated to an average size of 500 bp (30-s sonication, 30-s cooling for 10 cycles) using the Biorupter (Diagenode, Liege, Belgium). The sheared chromatin was mixed with 50 µL Protein G Sepharose 4 Fast Flow (catalog no. 1706 1801; GE Healthcare) for 1 h at 4 °C. After centrifugation at 10,000xg for 1 min at 4 °C, the pre-cleared chromatin (30 µL for ChIP-seq) was used as input. Chromatin aliquots (300 µL) for Esc2-4Flag or Rad57-4Flag ChIP were immunoprecipitated with 50 µL anti-Flag M2 affinity gel (catalog no. A2220; Sigma Aldrich,) at 4 °C overnight. Bead-bound chromatin was washed twice in L buffer (50 mM HEPES–KOH pH 7.5, 140 mM NaCl, 0.1% deoxycholate, 1% Triton X-100, 1 mM EDTA), twice in L buffer with additional 350 mM NaCl, once in wash buffer (10 mM Tris–Cl pH 8. 0, 250 mM LiCl, 0.5% deoxycholate) and twice in Tris–EDTA (TE) buffer (10 mM Tris–Cl pH 7.5, 1 mM EDTA). Protein-DNA cross-linking was broken in 250 µL elution buffer (10 mM Tris–Cl pH 8.0, 1 mM EDTA, 1% SDS, 150 mM NaCl, 5 mM dithiothreitol (DTT)) at 65 °C overnight. The input sample was mixed with 120 µL of elution buffer and incubated at 65 °C overnight. The DNA sample was diluted to 500 µL with TE buffer, mixed with 500 µL phenol:chloroform: isoamyl alcohol (25:24:1) and centrifuged at 12,000 rpm for 5 min at room temperature (RT) on a benchtop centrifuge. The upper layer (400 µL) was mixed with 40 µL 3 M sodium acetate, 5 µL 10 mg/ml glycogen, and 890 µL 100% ethanol (EtOH), incubated at -80 °C for 15 min and centrifuged at 14,000 rpm at 4 °C for 15 min on a benchtop centrifuge. The DNA pellet was washed with 70% EtOH, air dried, and dissolved in 50 µL TE buffer containing 1 µL 10 mg/ml RNase. After incubation at 37 °C for 5 min, the DNA solution was stored at − 20 °C until use.

ChIP–seq libraries were generated using the NEBNext ChIP-Seq Library Prep Kit for Illumina according to the manufacturer’s protocol (catalog no. E6240S; New England Biolabs) and sequenced on an Illumina NovaSeq 6000 system using the 150 base pair pair-end sequencing kit. Sequence analysis was performed by Novogene (Beijing, China). The sequencing data (DRA012865) were uploaded to the Galaxy web platform (https://usegalaxy.org) for data analysis^63^. ChIP–seq raw reads which were trimmed using trim-galore! V. 0.6.3 (Babraham Informatics, Cambridge, UK), was aligned to the *Saccharomyces cerevisiae* genome assembly (sacCer3) using Bowtie2 v.2.5.3^60^ to create BAM files. IP BAMs (Esc2-4F, Rad57-4F and no tag control) were analyzed with MACS2 callpeak v.2.2.9.1 to generate Bdg files^68^. To remove read data of non-specific binding to antibodies from the IP data, new bdg data was generated using MACS2 bdgcmp with the IP bdg data as the treatment sample and no tag control as the control sample^68^. The obtained bdg files were visualized using the Integrative Genomics Viewer (IGV) v.2.7.2^69^.

### ChIP-qPCR

ChIP sample preparation followed the method described in the ChIP-seq section. The following points were modified. Chromatin aliquots (200 µL) for histone H3 at K36 trimethylation (H3K36me3) were immunoprecipitated with 2 µg anti-H3K36me3 antibody (catalog no. ab9050; Abcam, Cambridge, UK) at 4 °C overnight. Chromatin aliquots (200 µL) for Flag epitope-tagging proteins (Esc2 and Rad57) were immunoprecipitated with 50 µL anti-Flag©M2 Affinity Gel (catalog no. A2220; Sigma-Aldrich, Burlington, MA, USA) at 4 °C 3 h. To precipitate immunoprecipitated histone H3K36me3 chromatin, a chromatin mixture was mixed with 10 µL Protein G Sepharose Fast Flow for 2 h at 4 °C. ChIP-qPCR was performed as previously described using the primers listed in Table S1. The LightCycler 480 SYBR Green I Master Mix (catalog no. 04707516001; Roche Life Science, Pleasanton, CA, USA) was used to calculate the percentage of input for ChIP. The mix contained 10 µL of 2 × LightCycler 480 SYBR Green I Master, 2 µL each of 5 µM PCR forward and reverse primers, and input and immunoprecipitated DNA samples (final volume 20 µL). Reactions were performed for 1 cycle of 10 min at 95 °C; 45 cycles of 20 s at 95 °C, 20 s at 53 °C, and 20 s at 72 °C; 1 cycle of 60 s at 65 °C and 1 s at 95 °C using LightCycler Nano (Roche Life Science). The percentage of input for ChIP was calculated according to the following formula: % recovery = 2^ [(Cq (input)-log_2_(dilution ratio of input sample)-Cq (IPed sample)] × 100. Cq (+ Ab) was calculated by (Cq (IP) /Cq (Input average). Cq (no Ab) was calculated by (Cq (no Ab IP) /Cq (Input average). Actual % recovery = % recovery (+ Ab) − % recovery (no Ab). Triple-independent experiments were performed. The coordinates of the PCR primers used are shown in Table S1.

### Transformation efficiency

The his5+ DNA fragments to use integration were amplified from a 10 ng template DNA (pFA6a-His3MX6)^70^ using the Expand Taq high-fidelity DNA polymerase (catalog number 04 738 250 001; Roche Diagnostics, Basel, Switzerland). Two species of his5+ DNA fragments were obtained from a 50 µL PCR reaction comprising 10 µL 10× Expand Taq reaction buffer, 200 μM dNTPs, 0.5 µM forward and reverse oligonucleotides as primers, and 1 U Expand Taq high-fidelity DNA polymerase. The PCR program was as follows: 120 s at 94 °C, 10× (10 s at 94 °C, 30 s at 55 °C, 120 s at 68 °C), 20× (10 s at 94 °C, 30 s at 65 °C, 120 s at 68 °C), and 15 min at 68 °C. The PCR products were purified using the Wizard SV Gel and PCR Clean-Up System (catalog number A9281; Promega, Madison, WI, USA). The primers used are listed in Table S1.

To transform yeast cells, 2 × 10^8^ cells were suspended in 100 µL of one-step buffer (0.2 M LiAc, 40% PEG, 100 mM DTT) containing his5 + DNA fragment (400 ng). The cell suspension was incubated at 42 °C for 1 h and plated on SC agar medium depleting histidine. After incubation at 30 °C for 3 days, the number of his + colonies was counted and transformation efficiency was calculated using the following formula: Transformation efficiency (%) = (colony number at position 1)/(colony number at position 2) × 100.

### RNA isolation and RT–PCR

Total RNA was isolated from log-phase yeast cells by using the RNeasy Mini Kit (catalog no. 70104; Qiagen). Reverse transcription from RNA to DNA and RT-PCR were performed by using a One Step SYBR PrimeScript PLUS RT-PCR Kit (catalog no. RR096A; Takara-Bio) according to the manufacturer’s protocols. RT-PCR was run for 1 cycle of 5 min at 42 °C; 1 cycle of 10 s at 95 °C; 40 cycles of 5 s at 95 °C, 20 s at 55 °C; 1 cycle of 1 s at 95 °C; 1 cycle of 15 s at 65 °C and 1 s at 95 °C using either a LightCycler 480 System II or LightCycler Nano (Roche Life Science). The amount of each mRNA was compared to the amount of *ACT1* mRNA. The PCR primers are listed in Table S1.

### Reverse transcription and PCR

The isolated total RNA was converted into DNAs by using the reverse transcriptase. Reverse transcription from RNA to DNA and RT-PCR were performed by using a PrimeScript™ II 1st strand cDNA Synthesis Kit by using the kit primer (Random 6 mers) (catalog no. 6210A/B; Takara-Bio) according to the manufacturer’s protocols. RT-PCR was run for 1 cycle of 10 min at 30 °C; 1 cycle of 60 min at 42 °C and 15 min at 70 °C using LightCycler Nano (Roche Life Science). The transcription of the target mRNA was confirmed by PCR using the synthesized DNA as a template. The primers used in this reaction are listed in Table S1. The PCR reaction was run on a 1.5% agarose gel and electrophoresed at 100 V. After staining the agarose gel with ethidium bromide, the DNA bands were visualized by UV irradiation.

### Statistics and reproducibility

The text and figure legends provide statistical details, including n, mean, and statistical significance. Error bars in experiments represent the standard deviation (SD) or standard error of the mean (SEM) from independent experiments or independent samples. All statistical analyses were performed using Excel (Microsoft); detailed information on the statistical methods used is provided in the figure legends. The number of independent experiments or biological replicates and *P* values (*P < 0.05, **P < 0.01, ***P < 0.001, ****P < 0.0001) are given in the individual figures. *P* < 0.05 was considered statistically significant. The immunoblots in the figures show a representative image of at least three independent experiments or biological replicates with similar results.

### Supplementary Information

Supplementary Legends.

Supplementary Table S1.

Supplementary Table S2.

Supplementary Table S3.

Supplementary Figure S1.

Supplementary Figure S2.

Supplementary Figure S3.

Supplementary Figure S4.

Supplementary Figure S5.

Supplementary Figure S6.

Supplementary Figure S7.

Supplementary Figure S8.

Supplementary Figure S9.

## Data Availability

The datasets generated during the current study are available in the DDBJ repository, DRA012864 (WGS) and DRA012865 (ChIP-seq).
