# Supplementary material for: The Ty1 retrotransposon harbors a DNA region that performs dual functions as both a gene silencing and chromatin insulator
Source: Sci Rep. 2024 Jul 18;14:16641. doi: 10.1038/s41598-024-67242-z (PMC11258251; doi:10.1038/s41598-024-67242-z)
Supplement: Supplementary file 1 — Supplementary Legends. [file 41598_2024_67242_MOESM1_ESM.docx]

**Supplementary figure legends**

**Fig. S1.** **The measurement of mRNA size from the *GAL10* promoter, and Flag-Venus expression depends on the *TDH3* promoter.** **A**. The mRNA originating from 5’-LTR was detected through PCR analysis. The DNA was then analyzed using electrophoresis on a 1.0% agarose gel. p*GAL10*: *GAL10* promoter. Control (Vector): PHM927 (Table S1). **B**. The immunoblot using anti-GFP antibody to detect Flag-Venus between in the presence or absence of *TDH3* promoter. p*TDH3*: *TDH3* promoter

**Fig. S2.** **Supplementary data of the GAGsi IR sequence in Ty1 elements.** **A**. the alignments of IR sequences in 32 Ty1 elements. **B**. The immunoblot using anti-GFP antibody to compare the expression levels of Flag-Venus between in wild-type and two IR loop-region mutants (1C and 3C). 1C: T465C in *GAG* gene. 3C: G463C, T465C, A467C in *GAG* gene.

**Fig. S3.** **Heterochromatin and the chromatin structure surrounding actively transcribed gene region in budding yeast. A** and **B**. The heterochromatin structures in two loci. MNase-seq data monitoring histone H3, H3K9Ac, H3K27Ac, H4K12Ac, H3K4me3, H3K36me3 and H3K79me3. The ChIP-seq data were adapted from Badjatia, N. et al., (2021) (Accession No.GSE151348 and GSE14927). ^22^. The visualization was performed by IGV ^72^. **A.** Subtelomere region (Chr. V-L). **B.** MAT alpha locus (Chr. III). **C** and **D**. Euchromatin structure in budding yeast. **C.** *HXK2* locus. **D.** *IDH1* locus.

**Fig. S4.** **Bisulfite-sequencing (BS-seq) analysis of GAGsi region.** The number on each sequence position at the IR structure (bottom strand) indicates the CT conversion rates (%). The average values were determined based on all CT conversion rates for each individual strand.

**Fig. S5.** **Supplemental cell growth data in SC and SC-His plates.** **A.** Schematic of gene screening to control GAGsi silencing (left cartoon).The plate assay to monitor cell growth in SC or SC-His plates. The HMY1605 strain and 4 EMS-treated mutant strains (**A**). The gene deletion strain harbored the GAGsi region upstream *HIS3* gene under the pSyn promoter (**B**).

**Fig. S6.** **Localization of Esc2 and Rad57 in Ty1 element.** Occupancy of Esc2-4Flag and Rad57-4Flag were summed over 31 Ty1 elements by ChIP-seq data. The *GAG* gene in Ty1 element begins at 0 bp along the X-axis. Black arrowhead: the IR site.

**Fig. S7.** **The measurements of mRNA by PCR analysis.** The mRNA was detected by PCR after conversion to DNA as shown in Fig. S1A. The positions of the primers used in this PCR assay were marked as A to E shown in the cartoon. **Primer position A~E.** PCR analysis using each primer position. The DNA fragments were subjected to analysis using agarose gel　electrophoresis with a concentration of 1.0%. IR-WT: wild-type IR sequence. IR-mt: mutated IR sequence. Control (Vector): PHM991 (Table S1).

**Fig. S8. The level of Ty1 mRNA is reduced in either *esc2*Δ or *rad57*Δ strain.** The mRNA level was measured against *ACT1* mRNA as a reference (=1) by RT-PCR. The results are average ± sd (n=3, independent experiments) and analyzed by unpaired t-test (two-tailed).

**Fig. S9.** **The general structure of the GAGsi plasmid integrated into *TRP1*::*ura3-1* (natMX).**

**Table S1. The yeast strains, plasmids and PCR primers used in this study.**

**Table S2.** The CT conversion rates of BS-seq on GAGsi region

**Table S3. The genes harboring novel mutations by EMS treatment**. **A**. Gene list harboring novel mutations compared with wild-type. **B**. Genes classified in DNA metabolism. **C.** TF colony number list.
