# Supplementary material for: The Ty1 retrotransposon harbors a DNA region that performs dual functions as both a gene silencing and chromatin insulator
Source: Sci Rep. 2024 Jul 18;14:16641. doi: 10.1038/s41598-024-67242-z (PMC11258251; doi:10.1038/s41598-024-67242-z)
Supplement: Supplementary file 2 — Supplementary Table S1. [file 41598_2024_67242_MOESM2_ESM.docx]

|  | **Table S1**. Budding yeast strains, plasmids, and primers were used in this study. |
| --- | --- |

| **Strain name** | **Genotypes** | **The figure** | **Reference** |
| --- | --- | --- | --- |
| BY4742 | *MATα his3∆ leu2∆1 met15∆0 ura3∆0* |  | 54 |
| HMY1462 | *MATα his3∆ leu2∆1 met15∆0 ura3∆0 TRP1::URA3-1 (natMX)* |  | This study |
| HMY1538 | *MATα his3∆ leu2∆1 met15∆0 ura3∆0 TRP1::URA3-1 (natMX) pTDH3-Flag-Venus-Tguo1 (URA3)* | Fig. 1C | This study |
| HMY1540 | *MATα his3∆ leu2∆1 met15∆0 ura3∆0 TRP1::URA3-1 (natMX) tGAG* (0.7 kb)-p*TDH3-*Flag*-Venus-*Tguo1 *(URA3)* | Fig. 1C | This study |
| HMY1541 | *MATα his3Δ leu2Δ1 met15Δ0 ura3Δ0 TRP1::URA3-1 (natMX) tGAG* (0.7-1.7 kb)-p*TDH3-*Flag*-Venus-*Tguo1 *(URA3)* | Fig. 1C | This study |
| HMY1542 | *MATα his3Δ leu2Δ1 met15Δ0 ura3Δ0 TRP1::URA3-1 (natMX) tGAG* (1.7 kb)-p*TDH3-*Flag*-Venus-*Tguo1 *(URA3)* | Fig. 1C | This study |
| HMY1559 | *MATα his3Δ leu2Δ1 met15Δ0 ura3Δ0 TRP1::URA3-1 (natMX)* p*GAL10-GAG* (1.7 kb)-p*TDH3-*Flag*-Venus-*Tguo1 *(URA3)* | Fig. 1E- G | This study |
| HMY1538 | *MATα his3∆ leu2∆1 met15∆0 ura3∆0 TRP1::URA3-1 (natMX) pTDH3-Flag-Venus-Tguo1 (URA3)* | Fig. 2E and 2F | This study |
| HMY1541 | *MATα his3Δ leu2Δ1 met15Δ0 ura3Δ0 TRP1::URA3-1 (natMX) tGAG* (0.7-1.7 kb)-p*TDH3-*Flag*-Venus-*Tguo1 *(URA3)* | Fig. 2E and 2F | This study |
| HMY1603 | *MATα his3Δ leu2Δ1 met15Δ0 ura3Δ0 TRP1::URA3-1 (natMX) tGAG* (0.7-1.7 kb) IR-stem mutant-p*TDH3-*Flag*-Venus-*Tguo1 *(URA3)* | Fig. 2E | This study |
| HMY1612 | *MATα his3∆ leu2∆1 met15∆0 ura3∆0 TRP1::URA3-1 (natMX) tGAG* (0.7-0.95 kb)-p*TDH3-*Flag*-Venus-*Tguo1 *(URA3)* | Fig. 2F | This study |
| HMY1613 | *MATα his3Δ leu2Δ1 met15Δ0 uraΔ0 TRP1::URA3-1 (natMX) tGAG* (0.7-1.25 kb)-p*TDH3-*Flag*-Venus-*Tguo1 *(URA3)* | Fig. 2F | This study |
| HMY1614 | *MATα his3∆ leu2∆1 met15∆0 ura3∆0 TRP1::URA3-1 (natMX) tGAG* (0.7-1.45 kb)-p*TDH3-*Flag*-Venus-*Tguo1 *(URA3)* | Fig. 2F | This study |
| HMY1615 | *MATα his3∆ leu2∆1 met15∆0 ura3∆0 TRP1::URA3-1 (natMX) tGAG* (0.7-0.8 kb)-p*TDH3-*Flag*-Venus-*Tguo1 *(URA3)* | Fig. 2F | This study |
| HMY1616 | *MATα his3Δ leu2Δ1 met15Δ0 ura3Δ0 TRP1::URA3-1 (natMX) tGAG* (0.7-1.7 kb)-p*TDH3-*Flag*-Venus-*Tguo1 *(URA3)* | Fig. 2F | This study |
| HMY1870 | *MATα his3Δ leu2Δ1 met15Δ0 ura3Δ0 TRP1::URA3-1 (natMX) tGAG* (0.7-1.7 kb)-p*TDH3-*Flag*-Venus-*Tguo1 *(URA3) esc2Δ::kanMX* | Fig. 2F | This study |
| HMY1871 | *MATα his3Δ leu2Δ1 met15Δ0 ura3Δ0 TRP1::URA3-1 (natMX) tGAG* (0.7-1.7 kb)-p*TDH3-*Flag*-Venus-*Tguo1 *(URA3) rad57Δ::kanMX* | Fig. 2F | This study |
| HMY1613 | *MATα his3Δ leu2Δ1 met15Δ0 uraΔ0 TRP1::URA3-1 (natMX) tGAG* (0.7-1.25 kb)-p*TDH3-*Flag*-Venus-*Tguo1 *(URA3)* | Fig. 4A | This study |
| HMY1613 | *MATα his3Δ leu2Δ1 met15Δ0 uraΔ0 TRP1::URA3-1 (natMX) tGAG* (0.7-1.25 kb)-p*TDH3-*Flag*-Venus-*Tguo1 *(URA3)* | Fig. 4B and 4C | This study |
| HMY1751 | *MATα his3∆ leu2∆1 met15∆0 ura3∆0 TRP1::URA3-1 (natMX) tGAG (0.7-1.25 kb) hairpin stem mutation -pTDH3-Flag-Venus-Tguo1 (URA3)* | Fig. 4B and 4C | This study |
| HMY1625 | *MATα his3∆ leu2∆1 met15∆0 ura3∆0 TRP1::URA3-1 (natMX) tGAG* (0.7-1.7 kb)-2nd *GAG* hairpin-p*TDH3-*Flag*-Venus-*Tguo1 *(URA3)* | Fig. 4E | This study |
| HMY1626 | *MATα his3∆ leu2∆1 met15∆0 ura3∆0 TRP1::URA3-1 (natMX) tGAG* (0.7-1.7 kb)-2nd *GAG* hairpin stem mutant -p*TDH3-*Flag*-Venus-*Tguo1 *(URA3)* | Fig. 4E | This study |
| HMY1605 | *MATα his3∆ leu2∆1 met15∆0 ura3∆0 TRP1::URA3-1 (natMX) tGAG (0.7-1.7 kb) -pSyn-HIS3-Tguo1 (URA3)* | Fig. 5A | This study |
| HMY1638 | *MATa his3∆ leu2∆1 met15∆0 ura3∆0 TRP1::URA3-1 (natMX) tGAG (0.7-1.7 kb) -pSyn-HIS3-Tguo1 (URA3) rpd3*∆::*kanMX* | Fig. 5A | This study |
| HMY1640 | *MATa his3∆ leu2∆1 met15∆0 ura3∆0 TRP1::URA3-1 (natMX) tGAG (0.7-1.7 kb) -pSyn-HIS3-Tguo1 (URA3) ies4*∆::*kanMX* | Fig. 5A | This study |
| HMY1642 | *MATa his3∆ leu2∆1 met15∆0 ura3∆0 TRP1::URA3-1 (natMX) tGAG (0.7-1.7 kb) -pSyn-HIS3-Tguo1 (URA3) rad57*∆::*kanMX* | Fig. 5A | This study |
| HMY1643 | *MATa his3∆ leu2∆1 met15∆0 ura3∆0 TRP1::URA3-1 (natMX) tGAG (0.7-1.7 kb) -pSyn-HIS3-Tguo1 (URA3) sir4*∆::*kanMX* | Fig. 5A | This study |
| HMY1644 | *MATa his3∆ leu2∆1 met15∆0 ura3∆0 TRP1::URA3-1 (natMX) tGAG (0.7-1.7 kb) -pSyn-HIS3-Tguo1 (URA3) esc2*∆::*kanMX* | Fig. 5A | This study |
| HMY1645 | *MATa his3∆ leu2∆1 met15∆0 ura3∆0 TRP1::URA3-1 (natMX) tGAG (0.7-1.7 kb) -pSyn-HIS3-Tguo1 (URA3) zds1*∆::*kanMX* | Fig. 5A | This study |
| HMY1646 | *MATa his3∆ leu2∆1 met15∆0 ura3∆0 TRP1::URA3-1 (natMX) tGAG (0.7-1.7 kb) -pSyn-HIS3-Tguo1 (URA3) ebs1*∆::*kanMX* | Fig. 5A | This study |
| HMY1647 | *MATa his3∆ leu2∆1 met15∆0 ura3∆0 TRP1::URA3-1 (natMX) tGAG (0.7-1.7 kb) -pSyn-HIS3-Tguo1 (URA3) nup120*∆::*kanMX* | Fig. 5A | This study |
| HMY1750 | *MATα his3Δ leu2Δ1 met15Δ0 ura3Δ0 TRP1::URA3-1 (natMX) tGAG (0.7-1.25 kb)-pTDH3-Flag-Venus-Tguo1 (URA3) esc2-4Flag-Tadh1 (LEU2)* | Fig. 5B | This study |
| HMY1756 | *MATα his3D leu2D1 met15D0 ura3D0 TRP1::URA3-1 (natMX) tGAG (0.7-1.25 kb) hairpin stem mutation -pTDH3-Flag-Venus-Tguo1 (URA3) esc2-4Flag-Tadh1 (LEU2)* | Fig. 5B | This study |
| HMY1757 | *MATα his3Δ leu2Δ1 met15Δ0 ura3Δ0 TRP1::URA3-1 (natMX) tGAG* (0.7-1.25 kb) *hairpin stem mutation-*p*TDH3-Flag-Venus-Tguo1 (URA3) rad57-4Flag-Tadh1 (LEU2)* | Fig. 5B | This study |
| HMY1758 | *MATα his3Δ leu2Δ1 met15Δ0 ura3Δ0 TRP1::URA3-1 (natMX) tGAG (0.7-1.25 kb)-pTDH3-Flag-Venus-Tguo1 (URA3) rad57-4Flag-Tadh1 (LEU2)* | Fig. 5B | This study |
| HMY1870 | *MATα his3Δ leu2Δ1 met15Δ0 ura3Δ0 TRP1::URA3-1 (natMX) tGAG* (0.7-1.7 kb)-p*TDH3-*Flag*-Venus-*Tguo1 *(URA3) esc2Δ::kanMX* | Fig. 5C and 5D | This study |
| HMY1871 | *MATα his3Δ leu2Δ1 met15Δ0 ura3Δ0 TRP1::URA3-1 (natMX) tGAG* (0.7-1.7 kb)-p*TDH3-*Flag*-Venus-*Tguo1 *(URA3) rad57Δ::kanMX* | Fig. 5C and 5D | This study |
| HMY1673 | *MATα his3∆ leu2∆1 met15∆0 ura3∆0 TRP1::URA3-1 (natMX)* 5'-LTR*-GAG* (1.7 kb) (*YLRWTy1-3*)*-TDH3pro-Flag-Venus-Tguo1* | Fig. 6C~F | This study |
| HMY1674 | *MATα his3∆ leu2∆1 met15∆0 ura3∆0 TRP1::URA3-1 (natMX)* 5'-LTR*-GAG* (1.7 kb) (*YLRWTy1-3*) hairpin stem mutation *-TDH3pro-Flag-Venus-Tguo1* | Fig. 6C~F | This study |
| HMY1559 | *MATα his3Δ leu2Δ1 met15Δ0 ura3Δ0 TRP1::URA3-1 (natMX)* p*GAL10-GAG* (1.7 kb)-p*TDH3-*Flag*-Venus-*Tguo1 *(URA3)* | Fig. S1A and S1B | This study |
| HMY1901 | *MATα his3Δ leu2Δ1 met15Δ0 ura3Δ0 TRP1::URA3-1 (natMX)* p*GAL10-GAG* (1.7 kb)- Flag*-Venus-*Tguo1 *(URA3)* | Fig. S1B | This study |
| HMY1767 | *MATα his3Δ leu2Δ1 met15Δ0 ura3Δ0 TRP1::URA3-1 (natMX) GAG (1.7 kb)* IR loop mutation (3C: G463C, T465C, A467C in *GAG)-pTDH3-Flag-Venus-Tguo1 (URA3)* | Fig. S2B | This study |
| HMY1768 | *MATα his3Δ leu2Δ1 met15Δ0 ura3Δ0 TRP1::URA3-1 (natMX) GAG (1.7 kb) hairpin loop mutation (1C: T465C in GAG)-pTDH3-Flag-Venus-Tguo1 (URA3)* | Fig. S2B | This study |
| HMY1542 | *MATα his3Δ leu2Δ1 met15Δ0 ura3Δ0 TRP1::URA3-1 (natMX) tGAG* (1.7 kb)-p*TDH3-*Flag*-Venus-*Tguo1 *(URA3)* | Fig. S2B | This study |
| HMY1613 | *MATα his3Δ leu2Δ1 met15Δ0 uraΔ0 TRP1::URA3-1 (natMX) tGAG* (0.7-1.25 kb)-p*TDH3-*Flag*-Venus-*Tguo1 *(URA3)* | Fig. S4 | This study |
| HMY1605 EMS treatment No. 1~3 | *MATα his3Δ leu2Δ1 met15Δ0 ura3Δ0 TRP1::URA3-1 (natMX) tGAG (0.7-1.7 kb) -pSyn-HIS3-Tguo1 (URA3)* EMS treatment *(HIS+)* | Fig. S5A | This study |
| HMY1664 | *MATa his3∆ leu2∆1 met15∆0 ura3∆0 TRP1::URA3-1 (natMX) tGAG (0.7-1.7 kb) -pSyn-HIS3-Tguo1 (URA3) srs2*∆::*kanMX* | Fig. S5B | This study |
| HMY1662 | *MATa his3D leu2D1 met15D0 ura3D0 TRP:: ura3-1 (natMX) tGAG (0.7-1.7 kb) -pSyn-HIS3-Tguo1 (URA3) elg1*∆::*kanMX* | Fig. S5B | This study |
| HMY1669 | *MATa his3D leu2D1 met15D0 ura3D0 TRP1::URA3-1 (natMX) tGAG (0.7-1.7 kb) -pSyn-HIS3-Tguo1 (URA3) rad51*∆::*kanMX* | Fig. S5B | This study |
| HMY1670 | *MATa his3D leu2D1 met15D0 ura3D0 TRP1::URA3-1 (natMX) tGAG (0.7-1.7 kb) -pSyn-HIS3-Tguo1 (URA3) rad52*∆::*kanMX* | Fig. S55B | This study |
| HMY1672 | *MATa his3∆ leu2∆1 met15∆0 ura3∆0 TRP1::URA3-1 (natMX) tGAG (0.7-1.7 kb) -pSyn-HIS3-Tguo1 (URA3) rad54*∆::*kanMX* | Fig. S5B | This study |
| HMY1667 | *MATa his3∆ leu2∆1 met15∆0 ura3∆0 TRP1::URA3-1 (natMX) tGAG (0.7-1.7 kb) -pSyn-HIS3-Tguo1 (URA3) rad55*∆::*kanMX* | Fig. S5B | This study |
| HMY1675 | *MATα his3D leu2D1 met15D0 ura3D0 TRP1 esc2-4Flag-Tadh1 (LEU2)* | Fig. S6 | This study |
| HMY1692 | *MATα his3∆ leu2∆1 met15∆0 ura3∆0 TRP1 rad57-*4Flag-Tadh1 (*LEU2*) | Fig. S6 | This study |
| HMY1673 | *MATα his3∆ leu2∆1 met15∆0 ura3∆0 TRP1::URA3-1 (natMX)* 5'-LTR*-GAG* (1.7 kb) (*YLRWTy1-3*)*-TDH3pro-Flag-Venus-Tguo1* | Fig. S7 | This study |
| HMY1674 | *MATα his3∆ leu2∆1 met15∆0 ura3∆0 TRP1::URA3-1 (natMX)* 5'-LTR*-GAG* (1.7 kb) (*YLRWTy1-3*) hairpin stem mutation *-TDH3pro-Flag-Venus-Tguo1* | Fig. S7 | This study |
| BY4742 | *MATα his3∆ leu2∆1 met15∆0 ura3∆0* | Fig. S8 | 54 |
|  |  |  |  |
|  |  |  |  |
|  |  |  |  |

| **Primers** | **Target gene** | **Sequence** | **Figure** | **Direction** |
| --- | --- | --- | --- | --- |
| HMP1540 | GAG-*TDH3* promoter junction for RT-PCR | 5'-AACGGTCTGACGGCACTGTAC-3' | Fig. 1E | Forward |
| HMP1541 | GAG-*TDH3* promoter junction for RT-PCR | 5'-GAGAATGGACCTATGAACTGATGG-3' | Fig. 1E | Reverse |
| HMP897 | *ACT1* gene for RT-PCR | 5'-TTCCAGCCTTCTACGTTTCCATCC-3' | Fig. 1E | Forward |
| HMP898 | *ACT1* gene for RT-PCR | 5'-CCAGCGTAAATTGGAACGACGTG-3' | Fig. 1E | Reverse |
| HMP1322 | Amplification of Flag-Venus (F-V) | 5'-ATGTCTAGAATGGACTACAAGGACGATGAC -3' | Fig. 2D | Forward |
| HMP1385 | Amplification of Flag-Venus (F-V) | 5'- TGCCGTCCTCCTTGAAGTCGATGC-3' | Fig. 2D | Reverse |
| HMP1410 | Amplification of GAGsi | 5'-ATGTCGACGCTCCCTCTCAATTCCTACCTA -3' | Fig. 2D | Forward |
| HMP1405 | Amplification of GAGsi | 5'-ATAAGCTTAGATACCCAGTAAAAGTCTCCA -3' | Fig. 2D | Reverse |
| YLRWTy1-3-upF1.1 | BS-seq analysis | 5'-TTTCTTTAGCAAGAAGCGTGAG-3' | Figs. 4A and S4 | Forward |
| YLRWTy1-3-inR0.1 | BS-seq analysis | 5'-CATTTGCGTCATCTTCTAACAC-3' | Figs. 4A and S4 | Reverse |
| YLRWTy1-3-upF4 | BS-seq analysis | 5'-CTAATAACCTTGGAAGAAATGAC-3' | Figs. 4A and S4 | Forward |
| YLRWTy1-3-inR1.4 | BS-seq analysis | 5'-CAACGGAATTTTGTTGTCTCATC-3' | Figs. 4A and S4 | Reverse |
| M13-47 | The region upstream IR sequence of GAGsi for ChIP-qPCR (primer position 1) | 5'-CGCCAGGGTTTTCCCAGTCACGAC-3' | Figs. 4B, 5B, and 5C | Forward |
| HMP1769 | The region upstream IR sequence of GAGsi for ChIP-qPCR (primer position 1) | 5'-AGGTAGGAATTGAGAGGGAGCGTCG-3' | Figs. 4B, 5B, and 5C | Reverse |
| HMP1384 | *Venus* region for ChIP-pPCR (primer position 2) | 5'-ATGTCTAGAATGGACTACAAGGACGATGAC-3' | Figs. 4B, 5B, and 5C | Forward |
| HMP1385 | *Venus* region for ChIP-pPCR (primer position 2) | 5'-TGCCGTCCTCCTTGAAGTCGATGC-3' | Figs. 4B, 5B, and 5C | Reverse |
| HMP1540 | *GAG - TDH3* promoter junction for RT-qPCR | 5'-AACGGTCTGACGGCACTGTAC-3' | Fig. 6B~D | Forward |
| HMP1541 | *GAG - TDH3* promoter junction for RT-qPCR | 5'-GAGAATGGACCTATGAACTGATGG-3' | Fig. 6B~D | Reverse |
| HMP1409 | PCR to amplify *GAG*- *TDH3* promoter -Flag-*Venus* region | 5'-ATGTCGACGGTAGCGCCTGTGCTTCGGTTA-3' | Fig. S1A | Forward |
| HMP2006 | PCR to amplify *GAG*- *TDH3* promoter -Flag-*Venus* region | 5'-ATGTCGACTTACTCGTCCATGCCGAGAGT-3' | Fig. S1A | Forward |
| HMP1409 | PCR to amplify transcription product as primer set A | 5'-ATGTCGACGGTAGCGCCTGTGCTTCGGTTA -3' | Fig. S7: Primer position A | Forward |
| HMP2006 | PCR to amplify transcription product as primer set A | 5'- ATGTCGACTTACTCGTCCATGCCGAGAGT -3' | Fig. S7: Primer position A | Reverse |
| HMP1660 | PCR to amplify transcription product as primer set B | 5'-AGAAATCTGAGTGATGAGAAGAATGAT -3' | Fig. S7: Primer position B | Forward |
| HMP2006 | PCR to amplify transcription product as primer set B | 5'- ATGTCGACTTACTCGTCCATGCCGAGAGT -3' | Fig. S7: Primer position B | Reverse |
| HMP1409 | PCR to amplify transcription product as primer set C | 5'-ATGTCGACGGTAGCGCCTGTGCTTCGGTTA -3' | Fig. S7: Primer position C | Forward |
| HMP1541 | PCR to amplify transcription product as primer set C | 5'-GAGAATGGACCTATGAACTGATGG-3' | Fig. S7: Primer position C | Reverse |
| HMP1660 | PCR to amplify transcription product as primer set D | 5'-AGAAATCTGAGTGATGAGAAGAATGAT -3' | Fig. S7: Primer position D | Forward |
| HMP1541 | PCR to amplify transcription product as primer set D | 5'-GAGAATGGACCTATGAACTGATGG-3' | Fig. S7: Primer position D | Reverse |
| HMP1512 | PCR to amplify transcription product as primer set E | 5'- ATTCTAGAATGGTGAGCAAGGGCGAGGAGC -3' | Fig. S7: Primer position E | Forward |
| HMP2006 | PCR to amplify transcription product as primer set E | 5'- ATGTCGACTTACTCGTCCATGCCGAGAGT -3' | Fig. S7: Primer position E | Reverse |
|  |  |  |  |  |
|  |  |  |  |  |
|  |  |  |  |  |
|  |  |  |  |  |
|  |  |  |  |  |

| **Plasmid** | **Genotypes** | **The figure** | **Reference** |
| --- | --- | --- | --- |
| PHM761 | *ura3-1* gene with p*URA3* + p*TEF1*-*nat*MX-t*TEF1* in pRS404 | Construction of HMY1462 strain | This study |
| PHM882 | p*TDH3*-Flag-*Venus*-Tguo1 in pRS406 | Construction of HMY1538 strain | This study |
| PHM887 | tGAG (0.7 kb)-p*TDH3*-Flag-*Venus*-Tguo1 in pRS406 | Construction of HMY1540 strain | This study |
| PHM888 | tGAG (0.7-1.7 kb)-p*TDH3*-Flag-*Venus*-Tguo1 in pRS406 | Construction of HMY1541 strain | This study |
| PHM889 | GAG (Full length: 1.7 kb)-p*TDH3*-Flag-*Venus*-Tguo1 in pRS406 | Construction of HMY1542 strain | This study |
| PHM927 | p*GAL10*- GAG (Full length: 1.7 kb)-p*TDH3*-Flag-*Venus* +t*ADH1* in pRS406 | Construction of HMY1559 strain and PCR control in Fig. S1A | This study |
| PHM934 | *tGAG* (0.7-1.7 kb)-p*TDH3-*Flag*-Venus-*Tguo1 in pRS406 | Construction of HMY1615 strain | This study |
| PHM950 | t*GAG* (0.7-1.7 kb) hairpin stem mutation -p*TDH3*-Flag-*Venus*-Tguo1 in pRS406 | Construction of HMY1603 strain | This study |
| PHM959 | tGAG (0.7-0.95 kb)-p*TDH3*-Flag-*Venus*-Tguo1 in pRS406 | Construction of HMY1612 strain | This study |
| PHM960 | *tGAG* (0.7-1.25 kb) -p*TDH3*-Flag-*Venus*-Tguo1 in pRS406 | Construction of HMY1613 strain | This study |
| PHM961 | *tGAG* (0.7-1.45 kb)-p*TDH3-*Flag*-Venus-*Tguo1 in pRS406 | Construction of HMY1614 strain | This study |
| PHM962 | *tGAG* (0.7-0.8 kb)-p*TDH3-*Flag*-Venus-*Tguo1 in pRS406 | Construction of HMY1615 strain | This study |
| PHM1023 | tGAG (0.7-1.25 kb) hairpin stem mutation *-*p*TDH3-*Flag-Venus-Tguo1 in pRS406 | Construction of HMY1751 strain | This study |
| PHM975 | *tGAG* (0.7-1.7 kb)-2nd *GAG* hairpin-p*TDH3-*Flag*-*Venus*-*Tguo1 in pRS406 | Construction of HMY1625 strain | This study |
| PHM978 | *tGAG* (0.7-1.7 kb)-2nd *GAG* hairpin stem mutant - p*TDH3-*Flag*-*Venus*-*Tguo1 in pRS406 | Construction of HMY1626 strain | This study |
| PHM952 | *tGAG (0.7-1.7 kb) -pSyn-HIS3-Tguo1* in pRS406 | Construction of HMY1605 strain | This study |
| PHM992 | *esc2* C-t *–* 4xFlag -Tadh1 in pRS405 | Construction of HMY1675 strain | This study |
| PHM993 | *rad57* C-t - 4xFlag -Tadh1 in pRS405 | Construction of HMY1692 strain | This study |
| PHM991 | 5'-LTR*-GAG* (1.7 kb) (*YLRWTy1-3*)*-pTDH3-*Flag-Venus-Tguo1 in pRS406 | Construction of HMY1673 strain | This study |
| PHM994 | 5'-LTR*-GAG* (1.7 kb) (*YLRWTy1-3*) hairpin stem mutation *-pTDH3-*Flag-Venus-Tguo1 in pRS406 | Construction of HMY1674 strain | This study |
| PHM1130 | p*GAL10*- GAG (Full length: 1.7 kb)-p*TDH3*-Flag-*Venus* +tADH1 in pRS406 | Construction of HMY1901 strain | This study |
| PHM1033 | *GAG (1.7 kb)* IR loop mutation (3C: G463C, T465C, A467C in *GAG)-*p*TDH3-*Flag-Venus-Tguo1 *(URA3)* in pRS406 | Construction of HMY1767 strain | This study |
| PHM1040 | *GAG (1.7 kb) hairpin loop mutation (1C: T465C in GAG)-pTDH3-Flag-Venus-Tguo1 (URA3)* in pRS406 | Construction of HMY1768 strain | This study |
