# Supplementary figures and images for: The Ty1 retrotransposon harbors a DNA region that performs dual functions as both a gene silencing and chromatin insulator
Source: Sci Rep. 2024 Jul 18;14:16641. doi: 10.1038/s41598-024-67242-z (PMC11258251; doi:10.1038/s41598-024-67242-z)

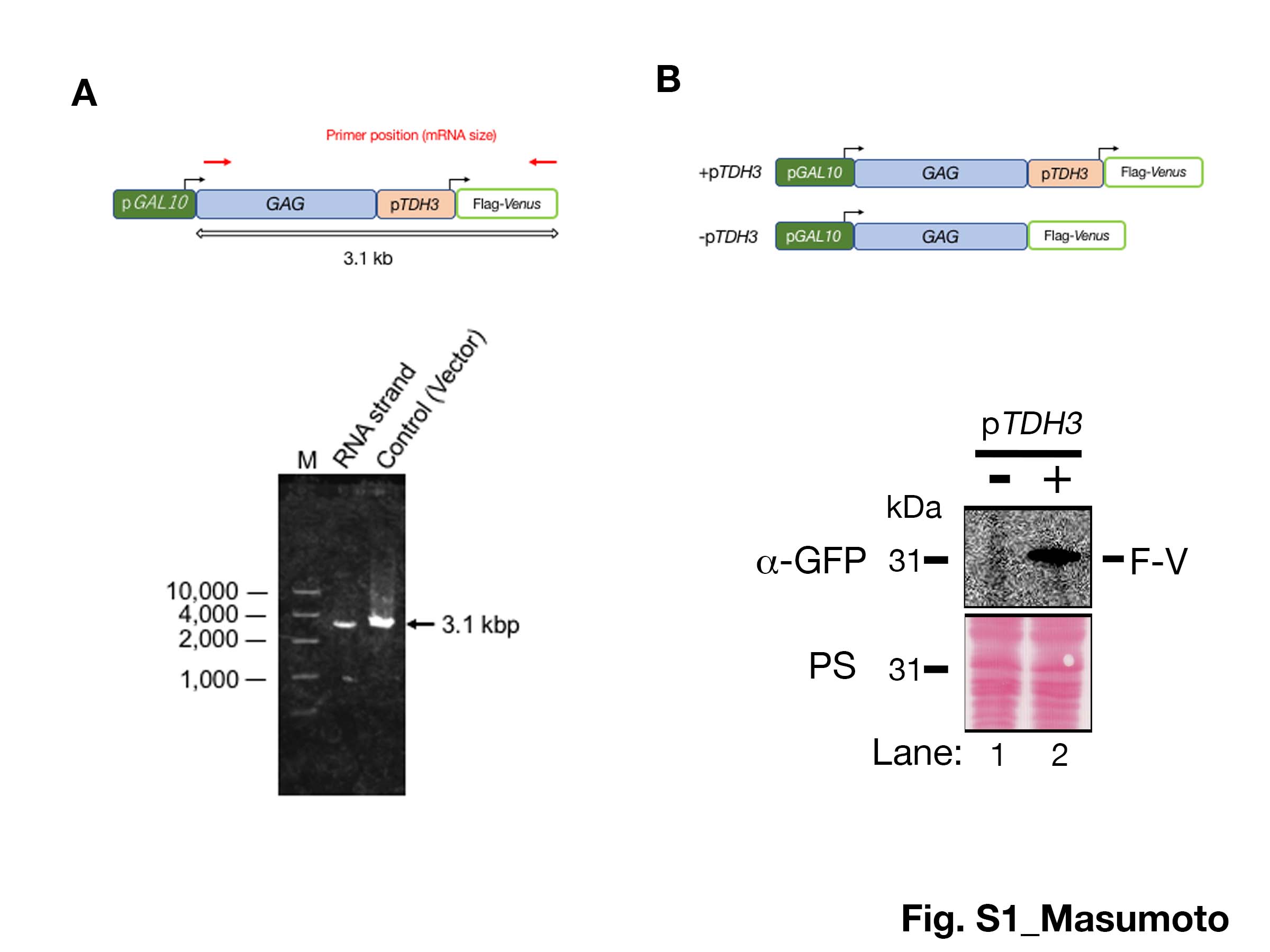

Supplement: Supplementary file 5 — Supplementary Figure S1. [file 41598_2024_67242_MOESM5_ESM.jpg]

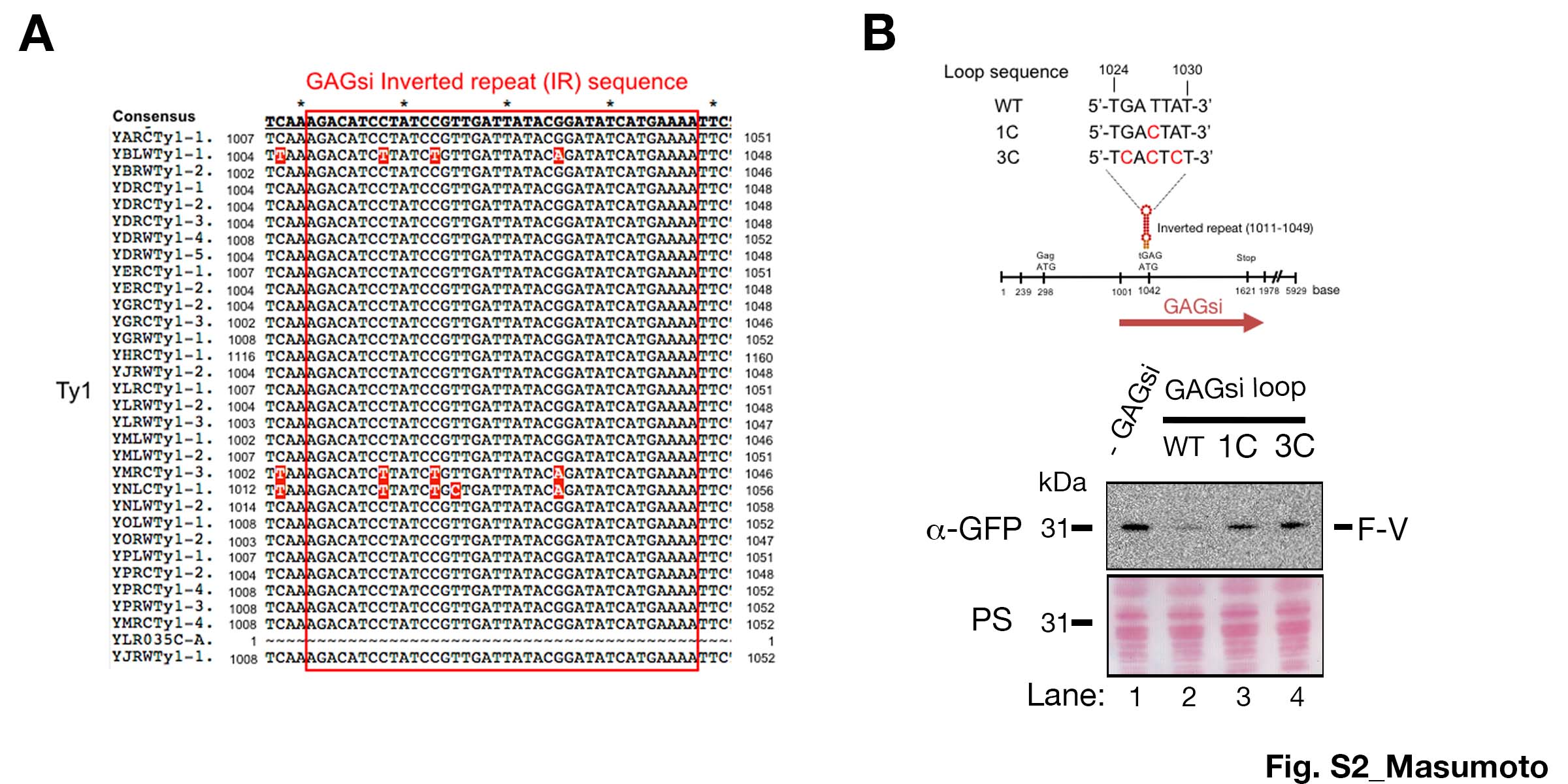

Supplement: Supplementary file 6 — Supplementary Figure S2. [file 41598_2024_67242_MOESM6_ESM.jpg]

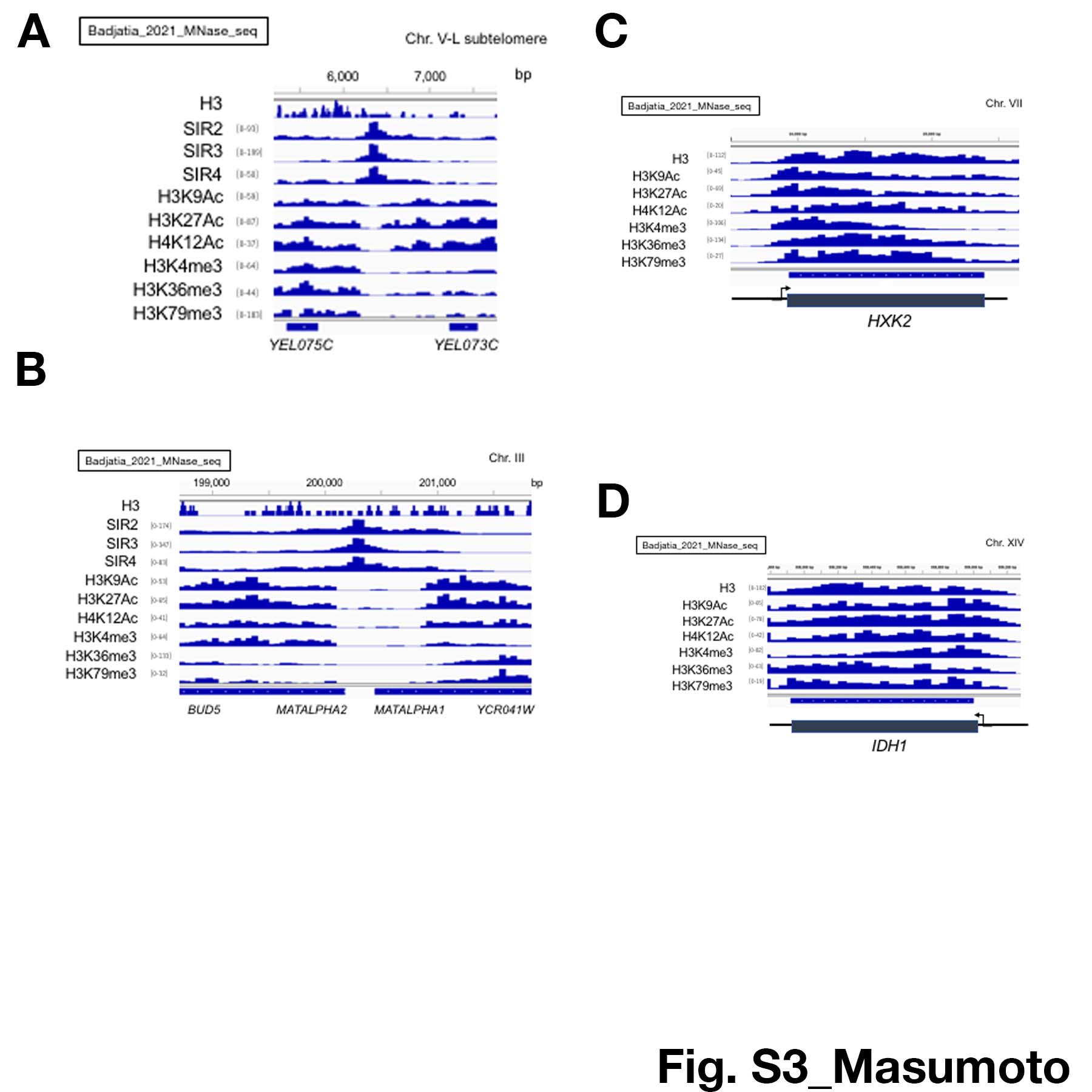

Supplement: Supplementary file 7 — Supplementary Figure S3. [file 41598_2024_67242_MOESM7_ESM.jpg]

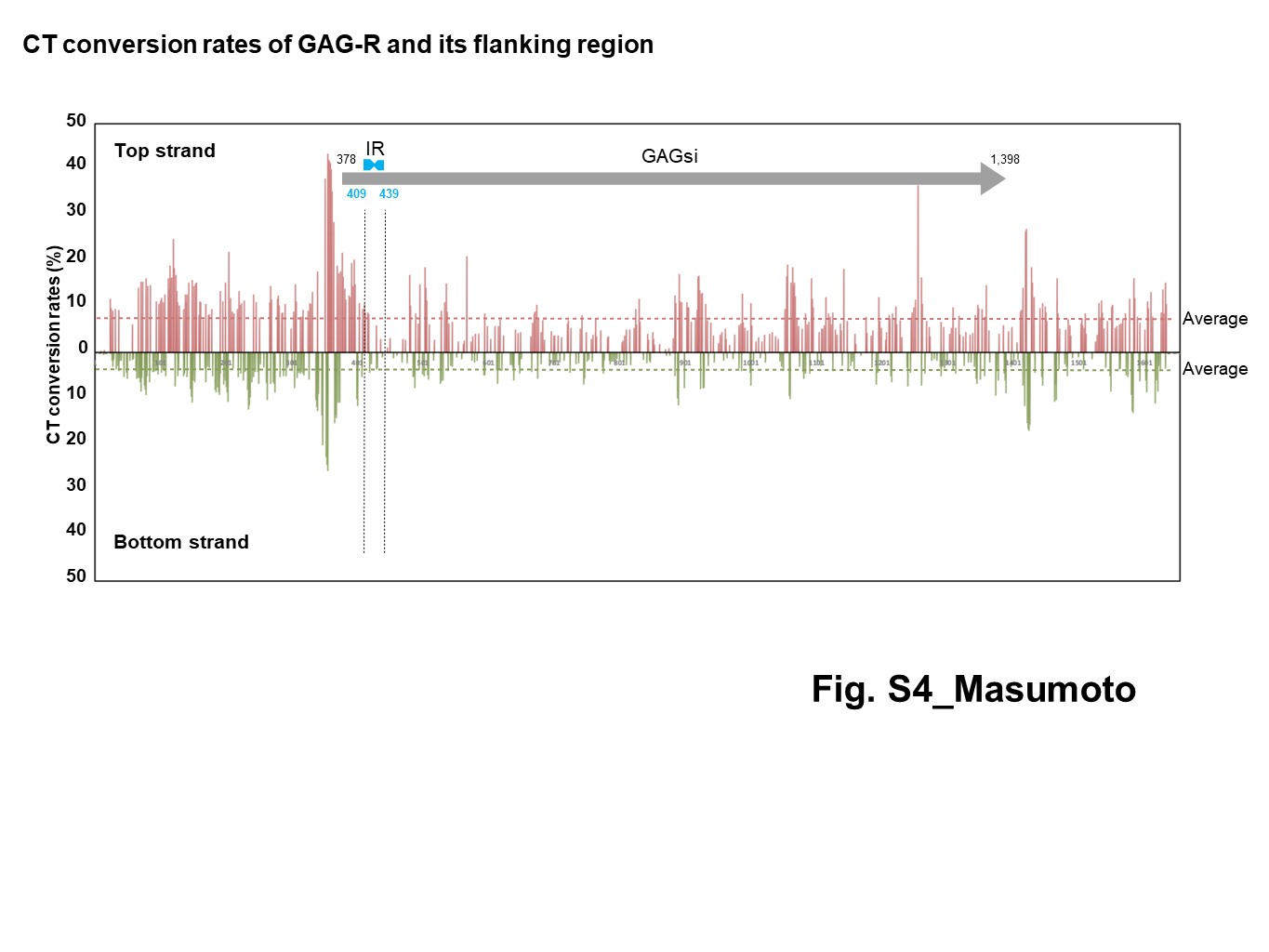

Supplement: Supplementary file 8 — Supplementary Figure S4. [file 41598_2024_67242_MOESM8_ESM.tif]

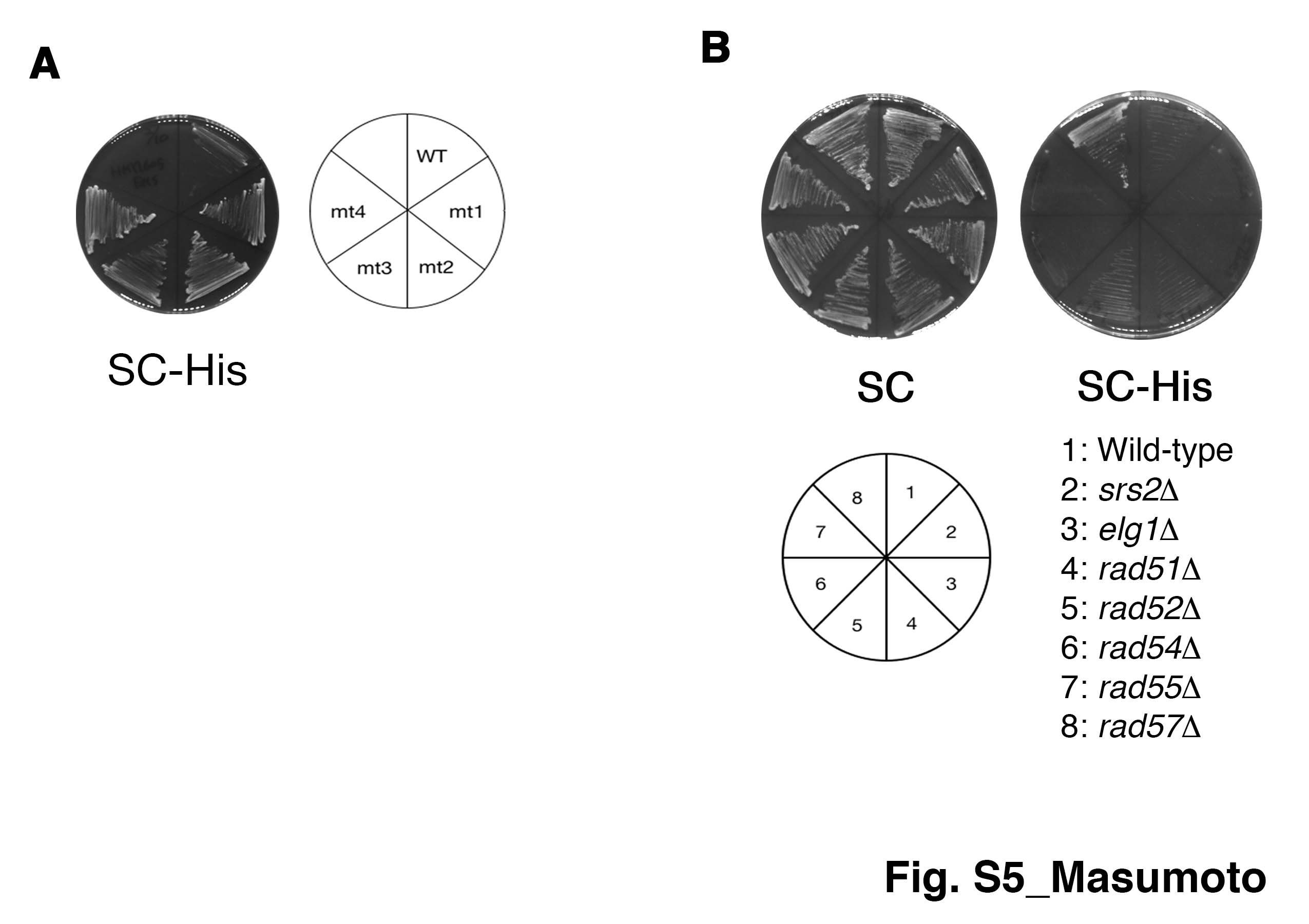

Supplement: Supplementary file 9 — Supplementary Figure S5. [file 41598_2024_67242_MOESM9_ESM.jpg]

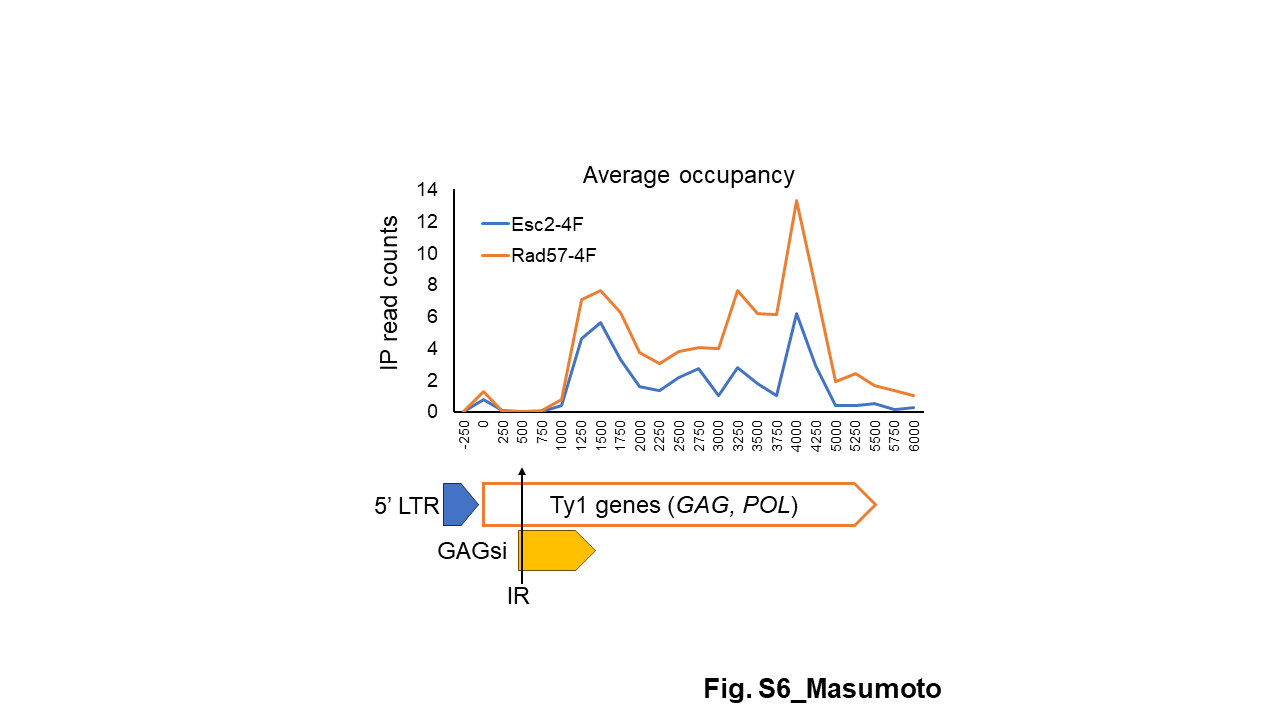

Supplement: Supplementary file 10 — Supplementary Figure S6. [file 41598_2024_67242_MOESM10_ESM.tif]

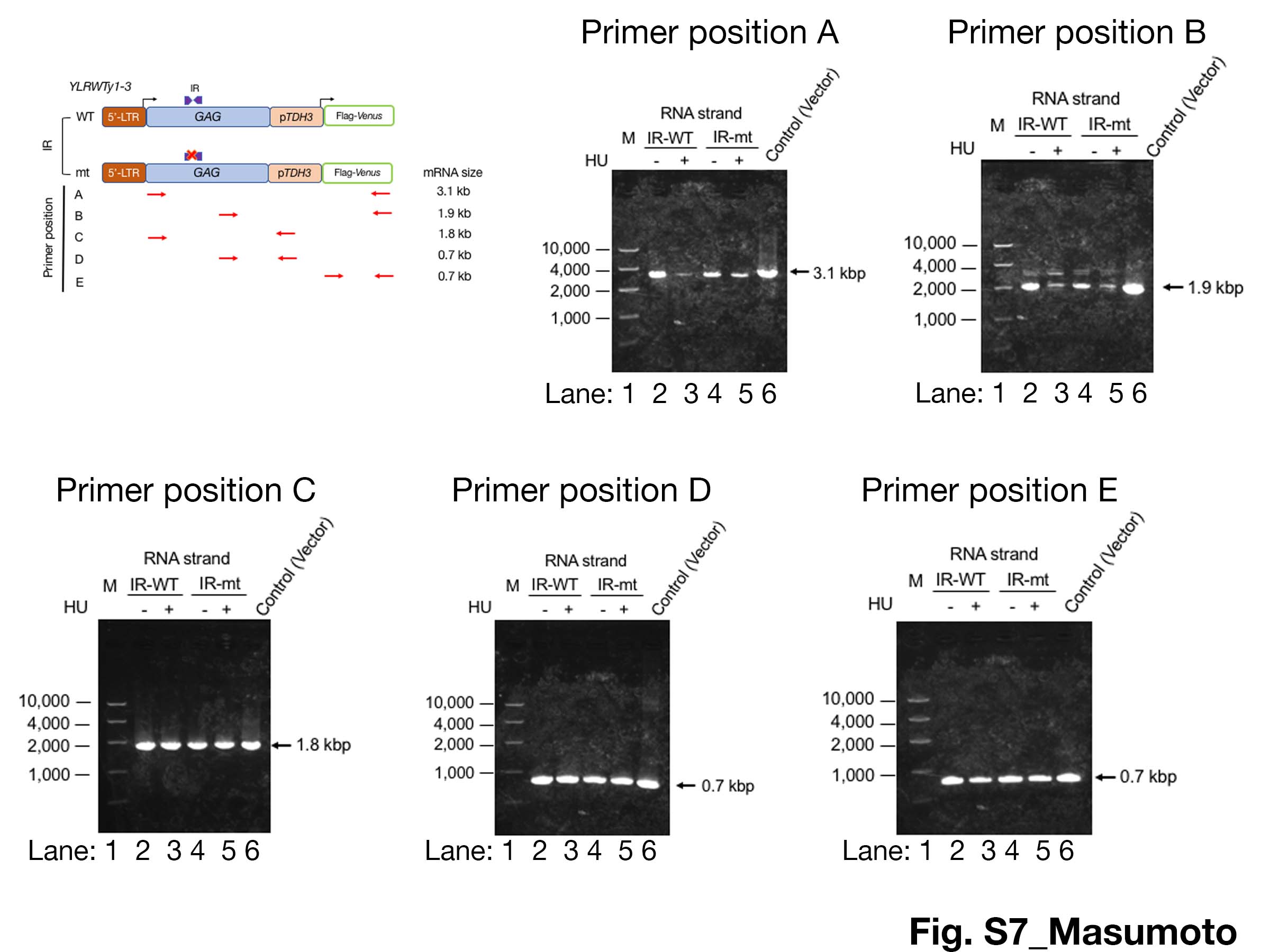

Supplement: Supplementary file 11 — Supplementary Figure S7. [file 41598_2024_67242_MOESM11_ESM.jpg]

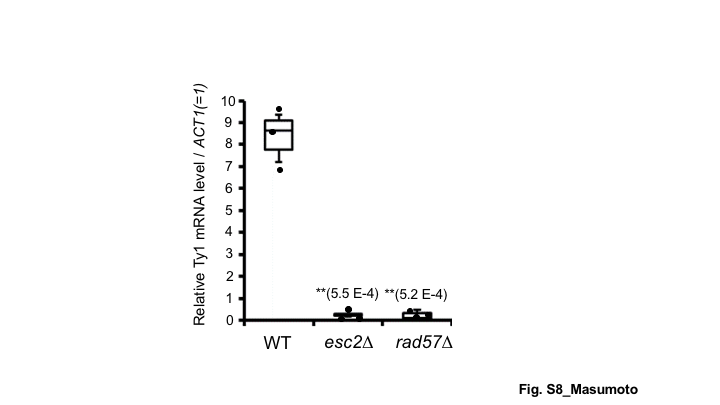

Supplement: Supplementary file 12 — Supplementary Figure S8. [file 41598_2024_67242_MOESM12_ESM.tiff]

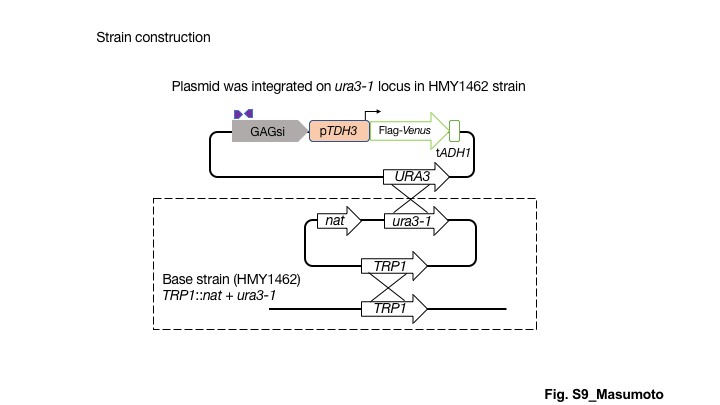

Supplement: Supplementary file 13 — Supplementary Figure S9. [file 41598_2024_67242_MOESM13_ESM.tiff]
